# Impact of patient sex on selection for abdominal aortic aneurysm repair: a discrete choice experiment

**DOI:** 10.1136/bmjopen-2024-091661

**Published:** 2025-02-26

**Authors:** Anna Louise Pouncey, Luis Enrique Loría-Rebolledo, Linda Sharples, Colin Bicknell, Mandy Ryan, Janet Powell

**Affiliations:** 1 Department of Surgery and Cancer, Imperial College London, London, UK; 2 Health Economics Research Unit, University of Aberdeen, Aberdeen, UK; 3 Medical Statistics, London School of Hygiene and Tropical Medicine, London, UK; 4 Institute of Applied Health Sciences, University of Aberdeen, Aberdeen, UK; 5 Imperial College London, London, UK

**Keywords:** Cardiovascular Disease, Vascular surgery, Health Equity

## Abstract

**Abstract:**

**Objectives:**

Women with an abdominal aortic aneurysm (AAA) are less likely to receive elective repair than men. This study explored the effect of patient sex and other attributes on vascular surgeons’ decision-making for infrarenal AAA repair.

**Design:**

Discrete choice experiment.

**Setting:**

Simulated environment using case scenarios with varying patient attributes.

**Participants:**

Vascular surgeons.

**Interventions:**

Surgical decision-making.

**Main outcome measures:**

AAA repair versus no repair and endovascular versus open repair.

**Results:**

182 surgeons completed 2987 scenarios. When all other attributes were equal, a woman was more likely to be offered an AAA repair (marginal rate of substitution (MRS) 3.86 (95% CI 2.93, 4.79)), while very high anaesthetic risk (MRS −4.33 (95% CI –5.63, –3.03)) and hostile anatomy (MRS −3.28 (95% CI –4.55, –2.01)) were deterrents. Increasing age did not adversely affect the likelihood of offering repair to men but decreased the likelihood for women, which negated women’s selection advantage from the age of 83 years. Women were also more likely to be offered endovascular repair (MRS 2.57 (95% CI 1.30, 3.84)).

**Conclusions:**

Patient sex alone did not account for real-world disparity observed in selection for surgery. Rather, being a woman was associated with a higher likelihood of being offered AAA repair but also a higher likelihood of being offered less invasive endovascular repair. Increased age decreased the likelihood of surgical selection for women but not men. Preference for less invasive repair, combined with inferior rates of anatomical suitability, and the comparably older age of women at the time of AAA repair selection may account for lower rates of repair for women observed.

**STRENGTHS AND LIMITATIONS OF THIS STUDY**

- The discrete choice experiment (DCE) design enabled evaluation of the relative importance of different attributes (eg, patient characteristics), which influence whether a surgeon decides to offer an abdominal aortic aneurysm (AAA) repair.
- The effect of attributes on AAA repair choices was consistent with real-world observations.
- Due to restrictions on the number of DCE attributes, not all variables which may be considered during selection for AAA repair could be included.
- This DCE examined surgeons’ preferences, which form only one component of the shared decision-making process.

## Introduction

 Despite a fourfold higher risk of rupture at smaller abdominal aortic aneurysm (AAA) diameters for women compared with men,^1 2^ previous research indicates that women with an AAA are up to 25% less likely to be offered elective surgical repair.^3 4^ Women are also less likely to be deemed morphologically suitable for minimally invasive endovascular aneurysm repair (EVAR), which has a lower operative mortality risk and is often tolerated by patients considered too frail to withstand the perioperative insult of open aortic repair (OAR).^35^,^7^

In the UK, this disparity in surgical selection is compounded by the availability of population-based AAA screening for men alone.^8^ As a result, although 1/5th of AAA diagnoses and 1/3rd of AAA ruptures are in women, only 1/10th of elective repairs are undertaken in women.^3 9 10^ Of the women who do receive surgery, an increased risk of mortality and complications, compared with men, for both elective OAR and EVAR is observed.^11^,^29^ This sex disparity in AAA repair and outcomes may arise from differential selection for surgery. For example, surgeons may elect to delay elective AAA repair for women until a later and more complex stage of their aneurysmal disease. Alternatively, it may reflect differences in comorbid status or anatomical features, which are associated with increased technical difficulty and adverse outcomes.^3 7 30 31^ While the NICE (National Institute for Health and Care Excellence) guidelines recommend the same diameter threshold for repair in men and women, other clinical practice guidelines recommend a lower threshold or acknowledge that the appropriate threshold for women is unknown.^32 33^

To understand why women are less likely to receive elective AAA repair, it is essential to examine whether and how sex or sex-specific patient attributes influence surgical selection. The surgeon’s opinion is a crucial part of the clinical decision-making process, which ideally includes input from a multidisciplinary team and shared decision-making with the patient.

This study employed a discrete choice experiment to investigate the factors influencing vascular surgeons’ treatment preferences for patients with AAA. Specifically, we examined two key decisions: (1) whether to recommend AAA repair or decline intervention (eg, ‘turn-down’) and (2) whether to opt for EVAR or open repair. The study aimed to explore whether patient sex impacts surgeons’ decision-making and to identify other factors that significantly influence their choices.

## Methods

A discrete choice experiment (DCE) is a choice-based survey which quantifies preferences for different options, such as treatment strategies. In these experiments, participants are presented with a series of clinical scenarios (known as choice sets), each defined by several attributes with specific levels (eg, age and sex).^34^ When DCE participants select the option they believe is best, their decision reflects how much they value the different features (or attributes) of that option.^35^,^37^ It relies on the assumption that people make rational choices, and when they do not, it is due to random factors. DCEs are used to produce a measure of benefit (or utility) for each attribute and level, helping to assess their relative importance and calculate the likelihood of different choices.^38^

Although the DCE method originated in economics,^35^,^37^ they are now increasingly used in healthcare to understand clinical decision-making.^39 40^ Relevant guidance from the ISPOR (International Society for Pharmacoeconomics and Outcomes Research) Conjoint Analysis Experimental Design Good Research Practices Task Force was used for this study.^41 42^ An extended description of methods and the distributed questionnaire are presented in online supplemental material, and a schematic of the DCE development process is available in online supplemental figure S1.

### Development of DCE design

To evaluate vascular surgeons’ decisions regarding (1) AAA repair and (2) selection for EVAR, a two-tier DCE was designed. The experiment presented a series of case scenarios featuring patients with AAA with varying attributes. Surgeons were asked to make two forced choices for each scenario: (1) AAA repair decision: Would you offer this patient AAA repair? (Yes/No) and (2) repair method decision: assuming this patient has been deemed suitable for repair, and could have an infrarenal clamp, would you prefer to offer endovascular rather than open repair? (Yes/No).

#### Selection of attributes and levels

The role of sex in AAA repair decision-making cannot be studied in isolation, as other factors influencing these decisions may also vary between women and men. Therefore, additional relevant attributes needed to be selected for the DCE. A multistage approach was used, starting with a scoping review of the literature to identify factors commonly used for AAA repair risk assessment and a review of current EVAR anatomical ‘Instructions For Use’ (IFU) criteria.^3343^,^65^ Next, interviews were conducted with consultant vascular surgeons,^66^ and qualitative analysis was performed to identify and prioritise attributes.^67^ This analysis followed the six-step process outlined by Clarke and Braun, using NVivo software (V.1.6.1, QSR International).^68^,^72^ Finally, a panel of six vascular surgeons reviewed the results to achieve consensus on attribute selection (defined as >75% agreement)^73 74^ (see online supplemental figure S1–S4).

#### Questionnaire design, pilot testing and distribution

A fractional factorial design with 16 scenarios was developed to create a choice set from which preferences could be estimated for all possible scenarios.^75^ The order of the scenarios was randomised to reduce the effect of participant fatigue. The design also included one additional scenario with clear dominance, to assess choice desirability, and one additional repeat scenario, to assess choice consistency.^76^ Participants provided informed consent and were blinded to the study’s primary objective. Additional data collected from participants included surgeon gender, level of experience (early: <10 years, mid-level: 10–20 years or experienced: >20 years), proportion of EVAR in clinical practice (categorised into 1st ‘low’, 2/3rd ‘mid-range’ and 4th ‘high’ quartiles) and geographical location (categorised as UK, Europe, North America or other).^77^,^80^ The survey was delivered using the Qualtrics XM Platform (Qualtrics, Provo, Utah, USA).

A pilot was then conducted with 18 vascular surgeons to assess whether the volume of scenarios was appropriate, assess respondent efficiency and gauge the perceived realism of the design.^81^ Pilot test results were also used to determine the minimum sample size requirement: 9 respondents completing 16 choice scenarios were needed to assess the effect of patient sex on selection for AAA repair and 140 respondents were needed for EVAR (α=0.05, β=0.2).^35^ Therefore, a recruitment target of 168 respondents was chosen, accounting for a 20% rate of attrition. The final selected attributes and levels are presented in table 1. Attributes for the Pilot test and full DCE are also presented within online supplemental table S1.

**Table 1:** Final selected attributes and levels for the discrete choice experiment

Final attribute	Levels
Patient sex	Man
	Woman
Age (years)	52, 64, 76 and 88
Anaesthetic risk	Anaesthetic risk=low
	Anaesthetic risk=moderate
	Anaesthetic risk=high
	Anaesthetic risk=very high
Patient perspective	The patient wants to have their aneurysm repaired
	The patient is worried about the risks of surgery
Abdominal aortic aneurysm size (mm)	50, 55, 60 and 65
Anatomy	Repair is on Instructions For Use
	Hostile neck will require adjuncts or complex repair
	Hostile access will require an adjunctive procedure
	Hostile neck will require adjuncts or complex repair and hostile access will require an adjunctive procedure

A multifaceted recruitment approach was employed, including international conferences, distribution via social media and collaboration through research networks. Participants were eligible if they were a consultant vascular surgeon with experience of both open and endovascular aortic repair and a good understanding of English. Prespecified withdrawal criteria included an incomplete survey response (eg, failure to consent or answering fewer than 50% of questions) and lack of clinical equipoise (eg, selecting the same alternative for all questions).

### Econometric analysis

Separate models were constructed for each outcome: (1) the likelihood of selecting AAA repair versus turndown and (2) the likelihood of selecting EVAR versus open repair. Each model included all relevant exposure variables: patient sex, age, aneurysm size, anatomical suitability, anaesthetic risk and patient perspective. The following utility/benefit function was estimated for each analysis using an error components model.

V_surgical_repair/endovascular_repair_ = ASC_0_ + ß_1_female_sex + ß_2_age + ß_3_anaes_mod + ß_4_anaes_high + ß_5_anaes_vhigh + ß_6_patient_anxiety + ß_7_AAA_diameter + ß_8_anatomy_neck + ß_9_anatomy_access + ß_10_anatomy_neck+access

V_turndown/open_repair_ = ASC_1_

Within this utility function, *V* represents the systematic utility—the predictable, measurable component of a decision based on known factors. The reference levels are (1) ‘turndown’ for analysis and (2) ‘open repair’ for analysis. The error components model was chosen to relax the irrelevance of independent alternatives assumption, classifying alternative specific constants as random: *ASC_0_* is set to 0, while *ASC_1_*, which captures the unexplained variation in choice for the reference levels, is modelled as a normally distributed random parameter and estimated using 500 Modified Latin Hypercube Sampling interindividual draws.^82^

Within the function, ß_1-10_ represents specific parameter coefficients associated with the defined attribute levels. Because utility is on a relative scale, the coefficients are interpreted in relation to each other rather than in absolute terms. A positive value indicates that the attribute level combination was preferred relative to the reference level, and a larger value indicates a stronger preference. To further aid interpretation, marginal rates of substitution (MRS), which enable assessment of trade-off between attributes, were calculated using AAA size (mm) as the denominator and the delta method for SE estimation.^83^ For instance, if a surgeon is deciding whether to offer a procedure based on a patient’s age and the size of their aneurysm, the MRS would indicate how much of an increase in AAA size would be needed to offset an increase in the patient’s age to keep the likelihood of offering the procedure the same. The mean percentage difference in the probability of selection for women and men was also calculated for a variety of scenarios.

All analyses were conducted in RStudio, using the Apollo R package.^84 85^ To improve model performance and aid interpretation, continuous data were centred and scaled, using the median value for age (70 years) and the threshold value (55 mm) for AAA size. Parameter estimates were presented with robust SEs, and a two-sided p value of <0.05 was considered significant. For each error components model, interactions between patient sex and other attributes were also introduced. The performance of models with interaction terms was compared using likelihood ratio, χ^2^ statistic and Akaike information criterion, and goodness of fit was estimated using adjusted pseudo R^2^. The assessment of final model performance was conducted using share prediction tests and fits tests, which assessed how the model’s predictions align with the actual observed data. Prespecified subgroup analyses were also conducted to assess preference heterogeneity associated with surgeon characteristics (eg, surgeon gender, level of experience, preference for EVAR repair and geographical location) reporting the proportion of observed choices compared with model predictions.

### Consistency, validity and sensitivity analyses

Sensitivity analyses were used to explore the effect of forced choice on evaluation of repair type selection by repeating the analysis for the subgroup of cases where ‘repair’ was selected in question A. The quality of choice data was also assessed using the following criteria: *Choice desirability*—a scenario where all patient-related features were set to their best creating a dominant choice, *Choice stability*—a repeated scenario to assess for consistency in responses and *Clinical Equipoise*—whether a participant selected the same choice for more than 90% of questions. Survey response time was used as an indicator of participant engagement. The stability of findings was evaluated through subgroup analyses excluding respondents who failed consistency and validity assessments.

## Results

A total of 182 respondents completed the DCE. AAA repair was chosen in 64.93% (1881/2897) and EVAR in 62.35% (1772/2842) of cases. 42 (23.08%) respondents were women, 70 (38.46%) had <10 years of experience, 69 (37.91%) had 10–20 years of experience and 36 (19.78%) had >20 years of experience. The median percentage of EVAR performed was 70 (IQR: 40–100). Geographically, 60 (32.97%) practiced in the UK, 48 (26.37%) in Europe, 45 (24.73%) in North America and others practicing in Latin America, the Middle East, South Africa and Southeast Asia (see online supplemental table S2).

### Selection for AAA repair

**Figure 1:**
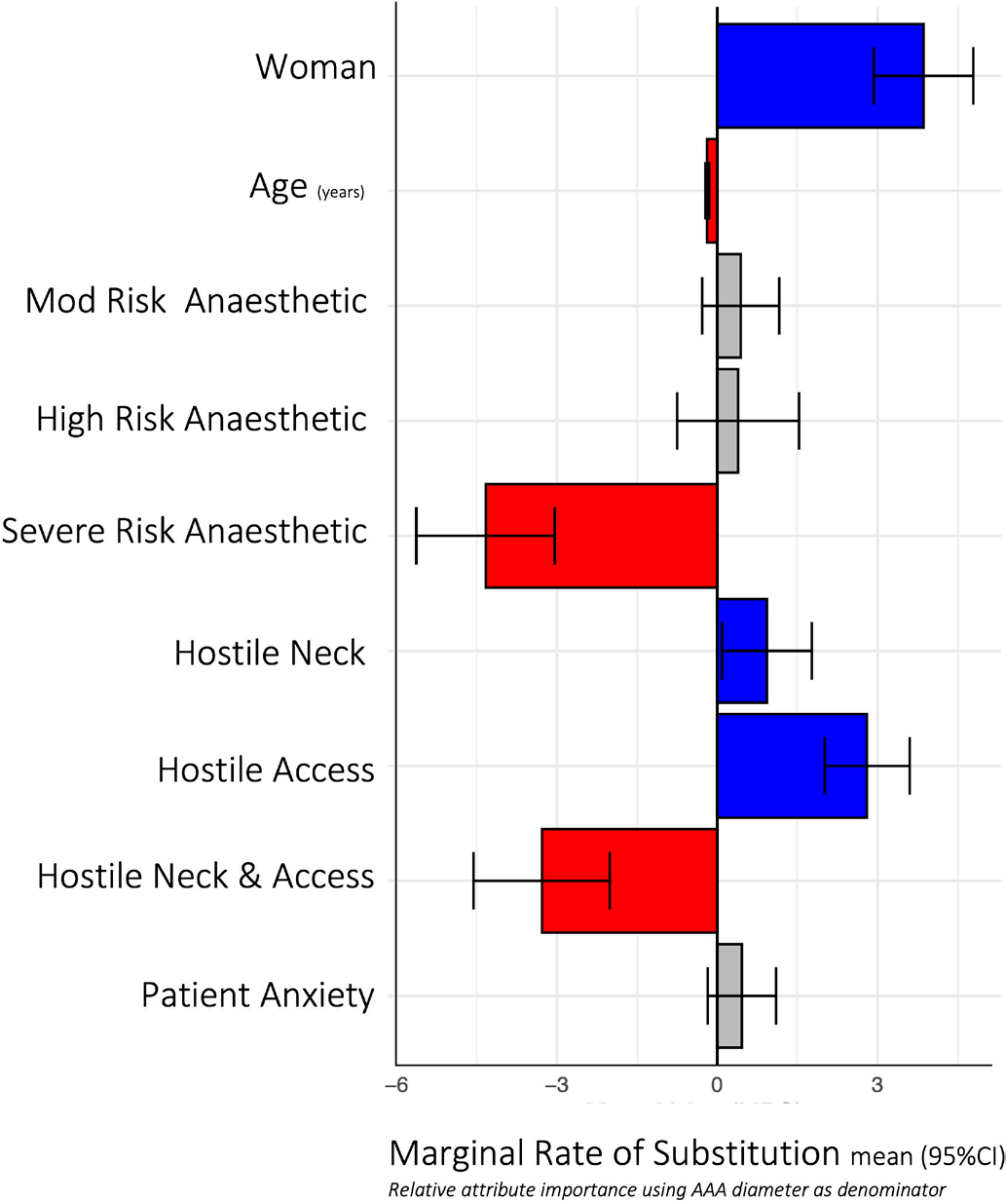
Marginal rates of substitution to enable evaluation of trade-off between attributes in relation to abdominal aortic aneurysm (AAA) size (mm) for selection for AAA repair (vs no repair). Blue=positive effect on the likelihood of selection for AAA repair, red=negative effect on the likelihood of selection for AAA repair, grey=no significant effect.

The error components model for selection for AAA repair (vs ‘turn-down’) is demonstrated in table 2. A woman patient and an increase in aneurysm size increased the likelihood of offering an AAA repair, while an increase in age, very high anaesthetic risk and hostile neck and access anatomy decreased the likelihood of offering an AAA repair. Patient-reported anxiety did not contribute to the surgeon’s decision-making. MRS in relation to AAA size indicated that being a woman had the greatest effect on increasing likelihood of repair selection (MRS 3.86 (95% CI 2.93, 4.79)), equivalent to a 3.86 mm increase in AAA size compared with men. Very high anaesthetic risk (compared with low anaesthetic risk) was the greatest deterrent (MRS −4.33 (95% CI –5.63, –3.03)), followed by the presence of hostile neck and access anatomy (compared with IFU anatomy, MRS −3.28 (95% CI −4.55, –2.01)). Increasing age was also associated with increased reluctance to offer repair (MRS −0.19 (95% CI −0.22, –0.15, per year)) (see figure 1).

**Table 2:** Error component model for AAA repair versus no repair

Parameter	Estimate	Robust SE	Robust t-ratio	Robust p value	Attribute MRS		
					Estimate	Lower 95% CI	Upper 95% CI
ASC no repair (mean)	−1.29	0.10	−12.8	<0.001	NA	NA	NA
ASC no repair (SD)	0.00	0.10	0.11	0.913	NA	NA	NA
Woman	1.12	0.11	9.86	<0.001	3.86	2.93	4.79
Age (years)	−0.05	0.01	−10.1	<0.001	−0.19	−0.22	−0.15
Anaesthetic risk: moderate	0.13	0.11	1.24	0.210	0.44	−0.28	1.16
Anaesthetic risk: high	0.12	0.17	0.69	0.490	0.39	−0.75	1.53
Anaesthetic risk: very high	−1.25	0.17	−7.55	<0.001	−4.33	−5.63	−3.04
AAA size (mm)	0.29	0.02	16.01	<0.001	NA	NA	NA
Hostile neck anatomy	0.27	0.13	2.15	0.030	0.93	0.09	1.77
Hostile access anatomy	0.81	0.12	6.57	<0.001	2.8	2.01	3.6
Hostile neck and access anatomy	−0.95	0.17	−5.52	<0.001	−3.28	−4.56	−2.01
Patient-reported anxiety	0.13	0.09	1.45	0.150	0.46	−0.18	1.1

Attribute reference levels: Mman, Aanaesthetic risk – low, Aanatomy on instructions for use (), patient wants procedure.

AAAabdominal aortic aneurysmASC, alternative specific constant; MRS, marginal rate of substitution, using AAA size as the denominator

### Selection for EVAR versus open repair

**Figure 2:**
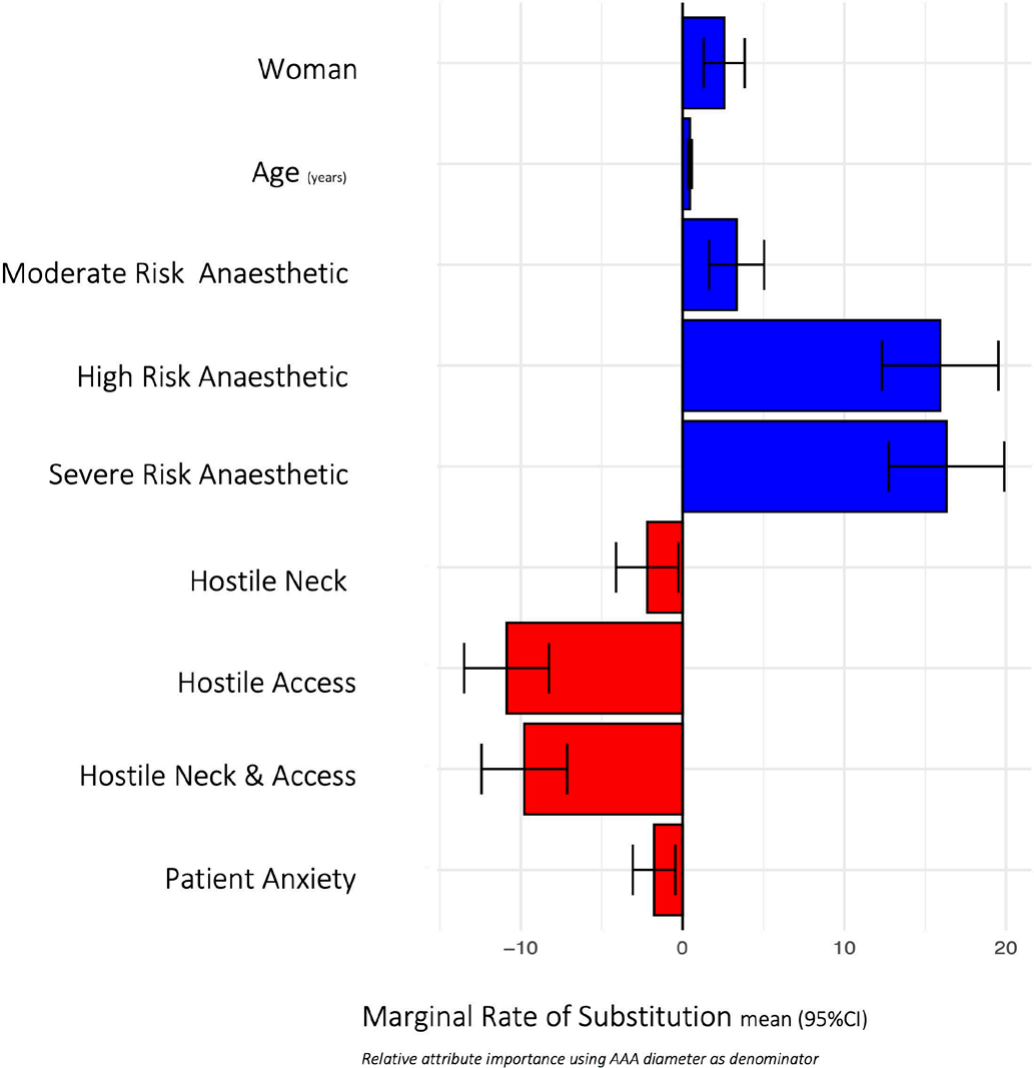
Marginal rates of substitution to enable evaluation of trade-off between attributes in relation to abdominal aortic aneurysm (AAA) size (mm) for selection for endovascular aneurysm repair (vs open repair). Blue=positive effect on the likelihood of selection for AAA repair, red=negative effect on the likelihood of selection for AAA repair, grey=no significant effect.

The error components model for selection for AAA repair method decision (EVAR vs open) is demonstrated in table 3. A woman patient, increased age, AAA size and anaesthetic risk were associated with an increased likelihood of selection for EVAR. Hostile neck and access anatomy and patient-reported anxiety decreased the likelihood of selection for EVAR. MRS in relation to AAA size (mm) indicated that very high anaesthetic risk, compared with low anaesthetic risk, had the greatest effect on the likelihood of offering EVAR rather than open repair (MRS 16.31 (95% CI 12.74, 19.89)). A woman patient (MRS 2.57 (95% CI 1.30, 3.84)) and increasing age also increased the likelihood of offering EVAR (MRS 0.45 (95% CI 0.34, 0.56), per year), while hostile access and neck anatomy decreased the likelihood of offering EVAR (MRS −9.79 (95% CI –12.43, –7.14)) (see figure 2).

**Table 3:** Error component model for EVAR versus open repair

Parameter	Estimate	Robust SE	Robust t- ratio	Robust p value	Attribute MRS		
					Estimate	Lower 95% CI	Upper 95% CI
ASC open (mean)	−1.37	0.12	−11.22	<0.001	NA	NA	NA
ASC open (SD)	0.00	0.10	0.09	0.930	NA	NA	NA
Woman	0.37	0.09	4.24	<0.001	2.57	1.3	3.84
Age (years)	0.06	0.01	10.59	<0.001	0.45	0.34	0.56
Anaesthetic risk: moderate	0.48	0.12	4.08	<0.001	3.34	1.64	5.04
Anaesthetic risk: high	2.27	0.18	12.38	<0.001	15.93	12.33	19.53
Anaesthetic risk: very high	2.33	0.18	12.92	<0.001	16.31	12.74	19.89
AAA size (mm)	0.14	0.01	13.12	<0.001	NA	NA	NA
Hostile neck anatomy	−0.31	0.14	−2.3	0.020	−2.19	−4.12	−0.26
Hostile access anatomy	−1.55	0.14	−11.03	<0.001	−10.88	−13.51	−8.26
Hostile neck and access anatomy	−1.40	0.16	−8.9	<0.001	−9.79	−12.43	−7.14
Patient-reported anxiety	−0.25	0.09	−2.67	0.010	−1.77	−3.08	−0.45

Attribute reference levels: Mman, Aanaesthetic risk – low, Aanatomy on instructions for use (), patient wants procedure.

AAAabdominal aortic aneurysmASC, alternative specific constant; EVAR, endovascular aneurysm repair; MRS, marginal rate of substitution, using AAA size as the denominator

### Attribute sex interactions

**Figure 3:**
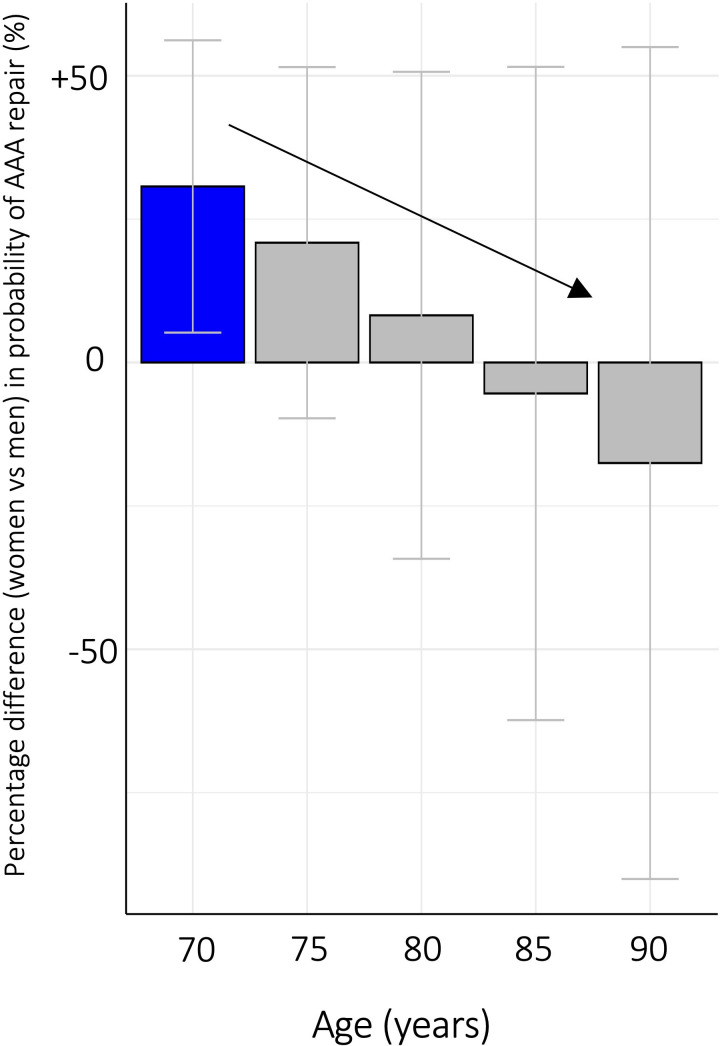
Percentage difference in the probability of selection for abdominal aortic aneurysm (AAA) for men and women using an error component model with an age-sex interaction term (see table 4). Blue=positive effect on the likelihood of selection for AAA repair, red=negative effect on the likelihood of selection for AAA repair, grey=no significant effect.

A significant interaction was observed between age and sex for selection for AAA repair. This model, demonstrated in table 4, performed better than the model without interactions. Within this age-sex interaction model, age had no significant effect on selection for AAA repair for men but was associated with a reduction in the likelihood of AAA repair for women, such that, all else being equal, the positive selection effect associated with being a woman was negated from the age of 83 years (see figure 3.) No significant interaction between sex and other attributes was observed to influence selection for EVAR.

**Table 4:** Error component model for AAA repair versus no repair, with patient sex-age interaction term

Parameter	Estimate	Robust SE	Robust t-ratio	Robust p value
ASC no repair (mean)	−1.42	0.11	−12.49	<0.001
ASC no repair (SD)	0.00	0.11	0.10	0.924
Woman	1.43	0.13	11.44	<0.001
Age (years)	−0.01	0.01	−1.17	0.240
Age: woman interaction	−0.11	0.01	−9.42	<0.001
Anaesthetic risk: moderate	−0.60	0.13	−4.48	<0.001
Anaesthetic risk: high	−0.58	0.17	−3.47	<0.001
Anaesthetic risk: very high	−1.38	0.14	−9.61	<0.001
AAA size (mm)	0.27	0.02	16.30	<0.001
Hostile neck anatomy	0.50	0.12	4.27	<0.001
Hostile access anatomy	1.12	0.13	8.61	<0.001
Hostile neck and access anatomy	−0.58	0.16	−3.54	<0.001
Patient-reported anxiety	0.11	0.10	1.14	0.250

Attribute reference levels: Mman, Aanaesthetic risk – low, Aanatomy on instructions for use (), patient wants procedure.

AAAabdominal aortic aneurysmASC, alternative specific constant

### Choice probabilities

**Figure 4:**
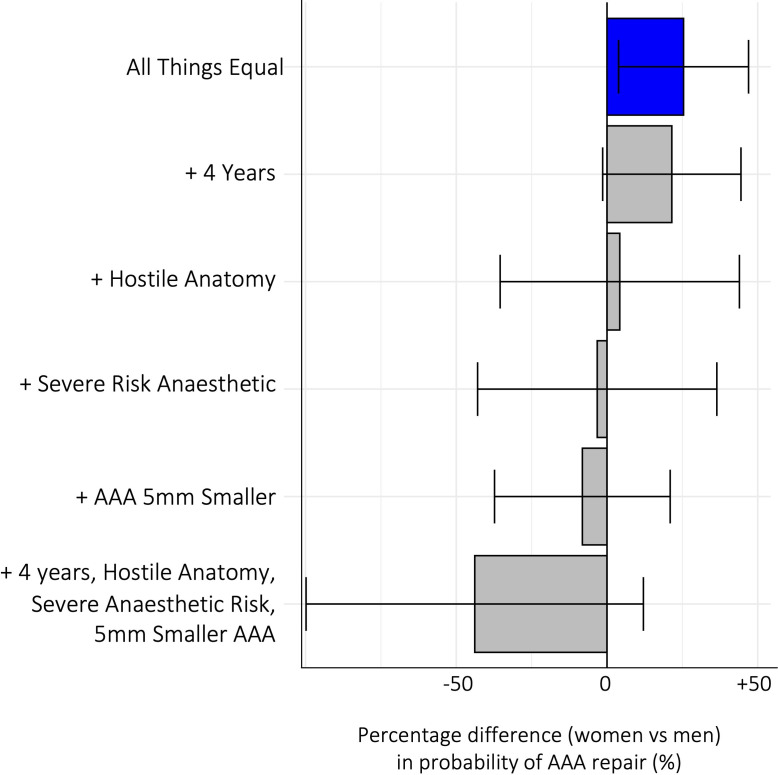
Percentage difference in probability of being offered abdominal aortic aneurysm (AAA) repair for women patients compared with men, using a main effects error component model (see table 3). Error bars=95% CI. Blue=positive effect on the likelihood of selection for AAA repair, red=negative effect on the likelihood of selection for AAA repair, grey=no significant effect.

When all other attributes were equal, women were significantly more likely to be offered AAA repair (percentage difference (%) 25.40 (95% CI 3.84, 46.96)); however, adjusting for real-world differences such as an increase in age of 4 years for women (21.50 (95% CI −1.44, 44.45)) or for hostile anatomy (4.24 (95% CI −35.45, 43.93)), the selection advantage for women was no longer significant. Moreover, adjusting for high anaesthetic risk (−3.25 (95% CI −42.93, 36.44)) or for an aneurysm 5 mm smaller in diameter (−8.18 (95% CI −37.31, 20.96)), women were less likely to be offered repair. A woman who was 4 years older, with hostile anatomy, high anaesthetic risk and a smaller aneurysm size (−5 mm) was 43.88% (95% CI –99.83, 12.08) less likely to be offered repair, compared with a man, with low anaesthetic risk, and anatomy within IFU (see figure 4).

### Respondent heterogeneity

No significant differences in selection for AAA repair, selection for EVAR or attribute importance were observed for men or women surgeons (132 men and 42 women). Early career surgeons offered AAA repair more frequently ((n=70) stated choice/model prediction (c/p): 777/725, p<0.001) than mid-level surgeons ((n=69) c/p: 646/710, p<0.001). Early career surgeons were more likely to offer EVAR to older patients than experienced surgeons (MRS age: early 0.62 (95% CI 0.44, 0.81), mid-level 0.40 (95% CI 0.24, 0.56) and experienced 0.21 (95% CI 0.02, 0.39)). No significant differences in the relative importance of other attributes were identified.

Surgeons in the lowest quartile for use of EVAR (n=31) were less likely to offer an AAA repair (c/p: 301/319, p<0.036) and less likely to offer EVAR (EVAR c/p: 236/313, p<0.001). Surgeons in the highest quartile for use of EVAR (n=54) were more likely to offer an AAA repair (c/p: 582/559, p<0.041) and more likely to offer EVAR (EVAR c/p: 536/632, p<0.001). Surgeons operating in the UK were less likely to offer an AAA repair ((n=60) c/p: 547/620, p<0.036) than surgeons in North America ((n=45) c/p: 514/466 p<0.001). UK surgeons were also less likely to offer EVAR (EVAR c/p: 521/599, p<0.001) than surgeons in North America (EVAR c/p: 495/452, p<0.001) (see online supplemental tables S3–S10).

### Assessment of consistency and validity

On assessment of choice desirability, 181/182 (99.45%) respondents chose to offer AAA repair for the dominant choice scenario. Most respondents demonstrated a degree of clinical equipoise: 6/182 (3.3%) offered AAA repair and 7/182 (3.9%) offered EVAR for over 90% of cases. Choice stability was superior for AAA repair for which 15/182 (8.24%) were inconsistent compared with EVAR for which 34/182 (18.68%) inconsistent. On subgroup analyses for those who met choice stability, choice desirability or equipoise or all three criteria, no significant difference in the direction of attribute effect or MRS was observed (see online supplemental tables 11 and 12).

## Discussion

This DCE investigated the effect of patient sex and additional factors on the likelihood of selection for AAA repair. Findings suggest that patient sex alone does not explain the observed disparity in clinician’s decision-making for AAA repair. Rather, when all other attributes were equal, a woman patient was more likely to be offered an AAA repair. However, on interaction analyses, while increased age had no significant effect for men, it reduced the likelihood of surgical selection for women. This negated the positive association with AAA repair selection for women from the age of 83 years. This finding has relevance as women are on average 4 years older than men at the time of AAA repair, and in hospital episode statistics data are reported to have an increased turndown rate at older ages, compared with men.^86 87^

Women were also more likely to be selected for less invasive EVAR. Preference for a less invasive approach, when combined with reduced anatomical suitability among women compared with men (34% vs 54%; OR 0·44 (95% CI 0.32, 0.62)), may therefore contribute to the lower rates of surgical selection for women reported.^3^ Indeed, the presence of hostile neck and access anatomy, when compared with IFU anatomy, was associated with lower likelihood of selection for both AAA repair and for EVAR and is demonstrated to be more common for women, even among patients selected for repair.^3 88^ Further, patient-related factors which were associated with reduced likelihood of selection for AAA repair and, conversely, increased likelihood of selection for less-invasive EVAR were age and increased anaesthetic risk. Of note, although women are often cited to have less comorbidity, they are more likely to have respiratory disease, which could translate into increased anaesthetic risk.^86^

Directions of attribute effect and heterogeneity in choice preferences were consistent with real-world observations. However, several limitations of DCEs to the healthcare setting have been cited, including normative issues, cognitive burden and applicability in different healthcare settings.^89^ Constraints on the number and complexity of DCE attributes meant that not all variables, such as the results of physiological fitness testing, could be included.^90^ Therefore, factors which have not been explicitly described may also influence selection for AAA repair. This analysis was restricted to infra-renal AAA, whereas in clinical practice, patients with hostile aortic neck anatomy may be considered for more complex repair options.^91^ Interpretation of findings is also limited by a lack of comprehensive real-world data regarding comorbid status for those not offered repair.^92^ The recruitment strategy may be biased towards opinions of those who engage in research and quality improvement. Additionally, variation in resources and standard practice may influence how respondents interpreted DCE attributes. Although study hypotheses were not disclosed within the survey, on-going research efforts by the team may have alerted respondents to the purpose of the study, potentially introducing bias towards offering AAA repair for women.

This DCE design examined surgeon’s preference, which forms only one part of the shared decision-making (SDM) process, and it is important to recognise that final treatment strategy also depends on patient and multidisciplinary opinions.^93^ The impact of SDM was examined in the OVIDIUS trial (Operative Vascular Intervention Decision-Making Improvement Using SDM tools) which included 73 men and 14 women with AAA. It found that patients were more likely to choose non-operative treatment (28.8% vs 21.4%) when SDM processes were used.^94^ However, whether and how priorities differ between women and men in SDM has been underreported. A recently published work to evaluate the effect of a decision aid on agreement between patient preference and AAA repair type included 234 men and only 1 woman.^95^ Further previous work, which sought to elicit AAA patient preferences, included only 5 women. However, 4 (80%) of these women expressed a preference for less invasive EVAR.^96^

Given the lack of prognostic data for women with AAA, surgeons may rely on sex stereotypes or personal experience to make operative decisions. A similar tendency towards less invasive treatment has been reported in cardiology, where women are less likely to receive complete revascularisation and considerably less likely to receive a coronary artery bypass grafting.^97 98^ Due to the lack of AAA screening for women, their AAAs are more often detected incidentally and their risk factors are less likely to be controlled. Combined with increased elective mortality rates, this may lead vascular surgeons to favour less invasive options.^86 99^ Additionally, the sex-frailty paradox—where women more often meet frailty criteria but experience lower mortality rates than men—may further increase preference for less invasive repair.^100^ However, these tendencies may also arise from a form of ‘benevolent sexism’ or ‘protective paternalism’, where a desire to protect women inadvertently results in a lower standard of care.^101 102^

## Conclusion

Patient sex alone did not account for real-world disparity observed in selection for surgery. Rather, being a woman was associated with a higher likelihood of being offered AAA repair but also a higher likelihood of being offered less invasive endovascular repair. Increased age decreased the likelihood of surgical selection for women but not men. Preference for less invasive repair, combined with inferior rates of anatomical suitability, and the comparably older age of women at the time of AAA repair selection may account for lower rates of repair for women observed.

## supplementary material

10.1136/bmjopen-2024-091661 online supplemental file 1

10.1136/bmjopen-2024-091661 online supplemental file 2

## Data Availability

Data are available upon reasonable request.
