# Supplementary material for: Impact of patient sex on selection for abdominal aortic aneurysm repair: a discrete choice experiment
Source: BMJ Open. 2025 Feb 26;15(2):e091661. doi: 10.1136/bmjopen-2024-091661 (PMC11865737; doi:10.1136/bmjopen-2024-091661)
Supplement: online supplemental file 1 [file bmjopen-15-2-s001.pdf]

Imperial College London

Default Question Block

This study aims to increase our understanding of how vascular surgeons decide whether or not to offer AAA repair, and also, whether to choose endovascular repair.

You will be presented with 18 scenarios for patients with an infrarenal AAA and will be asked to select:

(1) whether or not you would offer an AAA repair?

THEN assuming the patient will have an AAA repair:

(2) whether you would prefer to offer endovascular, rather than open, repair?

Please answer the questions as you would do in your normal practice. There are no right or wrong answers.

*****Please select a response to question 2 regardless of whether you offered repair, or not, in question 1.***

The study has been designed and is being conducted by the Imperial College Department of Surgery and Cancer. Should you have any questions regarding the study you may contact the research team on the following email: a.pouncey@imperial.ac.uk.

Further information is available to download here: participant information sheet.

It is expected that the survey will take less than 10-20 minutes to complete.

No identifying information will be collected and, following completion of the survey, there will be no follow-up or further tasks required.

I consent to take part in this study

Section 2

	Male
	52 years
	Anaesthetic risk = Low
	65mm infrarenal AAA
	Anatomy within IFU
	The patient would like their AAA repaired

	Yes	No
**(1) Would you offer an AAA repair to this patient?**	○	○
**(2) Would you prefer to offer endovascular repair?** Assuming the patient has been selected for surgery and could accommodate an infrarenal clamp.	○	○

	Female
AGE	52 years
	Anaesthetic risk = Moderate
	55mm infrarenal AAA
	Hostile access will require an adjunctive procedure
	The patient would like their AAA repaired

	Yes	No
**(1) Would you offer an AAA repair to this patient?**	○	○
**(2) Would you prefer to offer endovascular repair?** Assuming the patient has been selected for surgery and could accommodate an infrarenal clamp.	○	○

	Female
	88 years
	Anaesthetic risk = Very High
	50mm infrarenal AAA
	Hostile access will require an adjunctive procedure
	The patient is anxious about the risks of surgery

	Yes	No
**(1) Would you offer an AAA repair to this patient?**	○	○
**(2) Would you prefer to offer endovascular repair?** Assuming the patient has been selected for surgery and could accommodate an infrarenal clamp.	○	○

	Male
	88 years
	Anaesthetic risk = High
	55mm infrarenal AAA
	Anatomy within IFU
	The patient is anxious about the risks of surgery

	Yes	No
**(1) Would you offer an AAA repair to this patient?**	○	○
**(2) Would you prefer to offer endovascular repair?** Assuming the patient has been selected for surgery and could accommodate an infrarenal clamp.	○	○

	Male
	88 years
	Anaesthetic risk = Moderate
	60mm infrarenal AAA
	Hostile neck requiring adjuncts or complex repair
	The patient would like their AAA repaired

	Yes	No
**(1) Would you offer an AAA repair to this patient?**	○	○
**(2) Would you prefer to offer endovascular repair?** Assuming the patient has been selected for surgery and could accommodate an infrarenal clamp.	○	○

	Female
AGE	88 years
	Anaesthetic risk = Low
	65mm infrarenal AAA
	Hostile neck requiring adjuncts or complex repair<br>Hostile access will require an adjunctive procedure
	The patient would like their AAA repaired

	Yes	No
**(1) Would you offer an AAA repair to this patient?**	○	○
**(2) Would you prefer to offer endovascular repair?**<br>Assuming the patient has been selected for surgery and could accommodate an infrarenal clamp.	○	○

	Male
AGE	76 years
	Anaesthetic risk = Very High
	55mm infrarenal AAA
	Hostile neck requiring adjuncts or complex repair<br>Hostile access will require an adjunctive procedure
	The patient would like their AAA repaired

	Yes	No
**(1) Would you offer an AAA repair to this patient?**	○	○
**(2) Would you prefer to offer endovascular repair?**<br>Assuming the patient has been selected for surgery and could accommodate an infrarenal clamp.	○	○

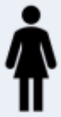	Female
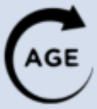	76 years
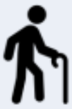	Anaesthetic risk = High
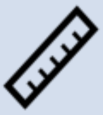	50mm infrarenal AAA
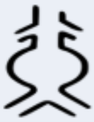	Anatomy within IFU
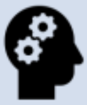	The patient would like their AAA repaired

	Yes	No
**(1) Would you offer an AAA repair to this patient?**	◯	◯
**(2) Would you prefer to offer endovascular repair?** Assuming the patient has been selected for surgery and could accommodate an infrarenal clamp.	◯	◯

	Female
	76 years
	Anaesthetic risk = Moderate
	65mm infrarenal AAA
	Anatomy within IFU
	The patient is anxious about the risks of surgery

	Yes	No
**(1) Would you offer an AAA repair to this patient?**	○	○
**(2) Would you prefer to offer endovascular repair?** Assuming the patient has been selected for surgery and could accommodate an infrarenal clamp.	○	○

	Male
AGE	76 years
	Anaesthetic risk = Low
	60mm infrarenal AAA
	Hostile access will require an adjunctive procedure
	The patient is anxious about the risks of surgery

	Yes	No
**(1) Would you offer an AAA repair to this patient?**	○	○
**(2) Would you prefer to offer endovascular repair?** Assuming the patient has been selected for surgery and could accommodate an infrarenal clamp.	○	○

	Female
	64 years
	Anaesthetic risk = Very High
	60mm infrarenal AAA
	Anatomy within IFU
	The patient would like their AAA repaired

	Yes	No
**(1) Would you offer an AAA repair to this patient?**	○	○
**(2) Would you prefer to offer endovascular repair?** Assuming the patient has been selected for surgery and could accommodate an infrarenal clamp.	○	○

	Male
	64 years
	Anaesthetic risk = High
	65mm infrarenal AAA
	Hostile access will require an adjunctive procedure
	The patient would like their AAA repaired

	Yes	No
**(1) Would you offer an AAA repair to this patient?**	○	○
**(2) Would you prefer to offer endovascular repair?** Assuming the patient has been selected for surgery and could accommodate an infrarenal clamp.	○	○

	Female
	64 years
	Anaesthetic risk = Moderate
	55mm infrarenal AAA
	Hostile neck requiring adjuncts or complex repair
	The patient is anxious about the risks of surgery

	Yes	No
**(1) Would you offer an AAA repair to this patient?**	○	○
**(2) Would you prefer to offer endovascular repair?** Assuming the patient has been selected for surgery and could accommodate an infrarenal clamp.	○	○

	Female
	64 years
	Anaesthetic risk = Moderate
	55mm infrarenal AAA
	Hostile neck requiring adjuncts or complex repair
	The patient is anxious about the risks of surgery

	Yes	No
**(1) Would you offer an AAA repair to this patient?**	○	○
**(2) Would you prefer to offer endovascular repair?** Assuming the patient has been selected for surgery and could accommodate an infrarenal clamp.	○	○

(repeat)

	Male
AGE	64 years
	Anaesthetic risk = Moderate
	50mm infrarenal AAA
	Hostile neck requiring adjuncts or complex repair<br>Hostile access will require an adjunctive procedure
	The patient is anxious about the risks of surgery

	Yes	No
**(1) Would you offer an AAA repair to this patient?**	○	○
**(2) Would you prefer to offer endovascular repair?** Assuming the patient has been selected for surgery and could accommodate an infrarenal clamp.	○	○

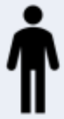	Male
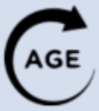	52 years
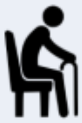	Anaesthetic risk = Very High
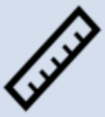	65mm infrarenal AAA
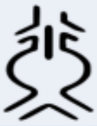	Hostile neck requiring adjuncts or complex repair
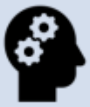	The patient is anxious about the risks of surgery

	Yes	No
**(1) Would you offer an AAA repair to this patient?**	○	○
**(2) Would you prefer to offer endovascular repair?** Assuming the patient has been selected for surgery and could accommodate an infrarenal clamp.	○	○

	Female
AGE	52 years
	Anaesthetic risk = High
	60mm infrarenal AAA
	Hostile neck requiring adjuncts or complex repair<br>Hostile access will require an adjunctive procedure
	The patient is anxious about the risks of surgery

	Yes	No
**(1) Would you offer an AAA repair to this patient?**	○	○
**(2) Would you prefer to offer endovascular repair?** Assuming the patient has been selected for surgery and could accommodate an infrarenal clamp.	○	○

Male
52 years
Anaesthetic risk = Low
50mm infrarenal AAA
Anatomy within IFU
The patient would like their AAA repaired

	Yes	No
**(1) Would you offer an AAA repair to this patient?**	○	○
**(2) Would you prefer to offer endovascular repair?** Assuming the patient has been selected for surgery and could accommodate an infrarenal clamp.	○	○

Section 3

What percentage of your AAA repairs are endovascular?

0 10 20 30 40 50 60 70 80 90 100

Percentage of endovascular AAA repair

What is your gender?

- ○ Male
- ○ Female
- ○ Non-binary / Other

How many years have you practised vascular surgery?

- ○ <10
- ○ 10-20
- ○ 20-30

In which country do you clinically practice?
