# Supplementary material for: Impact of patient sex on selection for abdominal aortic aneurysm repair: a discrete choice experiment
Source: BMJ Open. 2025 Feb 26;15(2):e091661. doi: 10.1136/bmjopen-2024-091661 (PMC11865737; doi:10.1136/bmjopen-2024-091661)
Supplement: online supplemental file 2 [file bmjopen-15-2-s002.pdf]

Supplemental Materials: The Impact of Patient Sex on Selection for Abdominal Aortic Aneurysm Repair: A Discrete Choice Experiment

SUPPLEMENTAL METHODS 3

Development of DCE Design 3

Identification and Selection of Attributes and Levels 4

Choice Set & Attribute Level Selection 19

Fractional Factorial Design of DCE 19

Survey Construction 19

Pilot Testing 20

Power Calculation 21

Administration of DCE Survey 21

Statistical Analysis 22

Econometric Analysis 22

Assessment of Consistency and Validity 24

Sensitivity Analysis 25

Supplemental Figures and Tables 26

Figure S1. 26

Figure S2. 27

Figure S3. 28

Figure S4. 29

Table S1. Pilot study and Final DCE Attributes and Levels. 30

Table S2. Summary of respondent characteristics. 31

Table S3. Surgeon gender subgroups: AAA repair versus no repair. 32

Table S4. Surgeon gender subgroups: EVAR versus open repair 33

Table S5. Surgeon experience level subgroups: AAA repair versus no repair. 34

Table S6. Surgeon experience level subgroups: EVAR versus open repair 35

Table S7. Use of EVAR in clinical practice subgroups: AAA repair versus no repair. 36

Table S8. Use of EVAR in clinical practice subgroups: EVAR versus open repair. 37

Table S9. Geographical location subgroups: AAA repair versus no repair. 38

Table S10. Geographical location subgroups: EVAR versus open repair 39

Table S11. Assessment of consistency & validity: AAA repair versus no repair. 40

Table S12. Assessment of consistency & validity: EVAR versus open repair. 41

SUPPLEMENTAL REFERENCES **42**

Supplemental Methods

The DCE method combines consumer theory and random utility theory, with experimental design theory and econometric analysis[1–3]. It relies on the key assumption that the subject will choose rationally, based on what they prefer, and when they do not, this can be explained by random factors. The basic description of this utility model is as follows:

$$U_{ij} = V_{ij} + \varepsilon_{ij}$$

where U represents utility, V represents the systematic observable component of the utility (e.g., choice set characteristics, defined by attributes,) and ε represents the unobservable stochastic component (e.g., measurement error or random preference). (Every alternative is indicated by j (j = 1, ..., J) and i denotes the consumer/user (i = 1, ..., I).) Therefore, when selecting an alternative with the maximum utility, the user selects the choice with the highest perceived value, which is determined, in part, by the perceived value of its attributes[1–3]. Relevant guidance from the ISPOR (International Society for Pharmacoeconomics and Outcomes Research) Conjoint Analysis Experimental Design Good Research Practices Task Force was utilised to ensure the appropriate design and conduct for a discrete choice experiment has been used for this study[4,5].

Development of DCE Design

To evaluate a vascular surgeon's decision for (A) AAA repair and (B) EVAR a two-tier DCE was designed. This consisted of a series of case scenarios presenting hypothetical AAA patients with varying attributes, for which two forced binary choices* were presented:

(A) Would you offer this patient AAA repair? - Yes/No

(B) Assuming this patient has been deemed suitable for repair, and could have an infrarenal clamp, would you prefer to offer endovascular rather than open repair? - Yes/No.

Identification and Selection of Attributes and Levels

The importance of sex cannot be studied in isolation as in reality women and men may also differ with regards to other anatomical and patient characteristics that also influence selection for AAA repair. Therefore, additional relevant attributes needed to be identified and selected for inclusion within the DCE. The identification and selection process for these attributes aimed to identify patient and anatomical characteristics and, to categorise and rank order them by importance, with special consideration regarding realistic thresholds and factors which may vary by sex (e.g., pertinent differences in aneurysm morphology such as access vessel diameter). A multistage approach including a scoping review of the literature, qualitative expert interviews with coding and thematic analysis, and an iterative selection process with a panel of vascular surgeons, were used to determine choice set selection and attribute levels. (See Figure S1.)

Scoping Review of the Literature

First a scoping review of the relevant literature, as well as national and international quality improvement and guidance standards was performed to identify attributes previously utilised in risk assessment for AAA repair[6–29]. From this review a summary of commonly used attributes for risk scoring systems were identified**Error! Reference source not found.**[10–13,15–29]. To examine morphological characteristics which might guide decision making between open repair and EVAR, the anatomical "Instructions For Use" (IFU) criteria, were also obtained for all currently approved devices for infrarenal EVAR. These findings were utilised to inform basic frameworks for the qualitative interview and for deductive coding, as well as for selection of relevant and realistic attribute levels, as outlined below.

Qualitative Assessment of Interview Data

Face-to-face, semi structured, preliminary “expert interviews” were conducted with a gender- and sex-balanced group of consultant vascular surgeons, (6 men and 5 women,) with experience of performing AAA repair at a major aortic centre, within the United Kingdom (UK), Europe (e.g., France) and the United States of America (USA). All surgeons performed a combination of open and endovascular procedures, with 7 stating predominantly mixed practice, 2 surgeons specialising predominantly in open and 2 specialising predominantly in endovascular techniques; and duration of clinical experience ranged from 15 – 30+ years. Qualitative analysis was used to identify and prioritise attributes, and to clarify attribute wording and interpretation for development of the DCE[30]. Interviews and thematic analyses were conducted until saturation of thematic identification was reached[31].

During consent for the interview, recording and analysis of transcripts, the following explanation was provided: “This interview aims to encourage the vascular surgeons to discuss their beliefs and offer advice about which factors are most important during surgical selection for open or endovascular AAA repair. We will use this to explore surgical decision making for men and women with an AAA and provide information to guide construction of a discrete choice experiment.”

Interviews were conducted using the following general framework for *open questioning*, which allowed the interviewee to then direct discussion and to talk in more depth about areas of their choosing[32]. Participant feedback was requested for each interview, and an iterative approach to the interview structure was employed, leading to the addition of question 5.

Questions to identify attributes for selection in the DCE:

1. What factors do you consider when selecting whether to offer a patient an open or endovascular AAA repair (or not)?

2. In the MDT setting, which factors do you like to see presented &, which are most important to guide you when forming an opinion about whether to offer surgery, and decide regarding the type of surgery?

Questions to identify attributes for which prevalence, levels or effect may vary by sex:

3. Have you noticed any differences in the risk factors typically found in men and women with an AAA?

4. Are there any patient or anatomical factors you feel are more important to consider pre-operatively when deciding whether to offer surgery for men or women?

Additional question incorporated following participant feedback:

5. Does cost play a role in your decision making for AAA repair?

Coding and Thematic Analysis

Thematic analysis entails in-depth and systematic review of qualitative data for a 'patterned response or meaning', which can be either *semantic,* addressing explicit meanings, or *latent,* reflecting deeper underlying meanings assumptions or ideologies[33].

For this study, thematic analysis was conducted within NVIVO software, (version 1.6.1, QSR International Pty Ltd.) and utilised a combined methodological approach[34,35]. First a *deductive* method, utilising the framework established from the scoping review of the literature was used to explore (a) the importance and characteristics of attributes utilised in decision making, and (b) attributes which may vary according to patient sex. Secondly, an *inductive* approach, was utilised to explore the surgeons' beliefs, further unanticipated attributes which may need to be consideration during DCE analysis. This secondary analysis could include identification of themes which may not be directly and explicitly expressed[34,35].

Analysis was conducted in accordance with the widely used six step process outlined by Clarke and Braun[33]. These steps are as follows: familiarising yourself with the data,

generating initial codes, searching for themes, reviewing themes, defining, and naming themes, and producing the report. This is a recursive process, allowing repetition of steps to clarify thematic codes and to re-visit the data considering new or emerging themes[33]. Code and word searches, cross-case comparisons, and hierarchy charts, available within NVIVO software, were used to further explore construct validity, cross-case reliability, and whether interpretation of the data had met the threshold of theoretical validity[31,36].

Qualitative Assessment of Vascular Surgeon's Interviews

Relatively minimal heterogeneity was observed in themes and sub-themes expressed according to surgeon gender, centre or country. Factors related to surgical decision making were broadly classified into six main themes: (1) patient-related factors, (2) aneurysm-related factors, (3) properties of the procedure, (4) surgeon-related factors - belief's, values, and expertise, (5) multidisciplinary team opinion and (6) shared decision-making. Variations in attributes and relative importance according to patient sex were also explored. (See Figures S2 and S3.)

Patient Age

The age of the patient was consistently identified as one of the most important factors utilised in surgical decision making but was rarely used in isolation or as an absolute threshold.

"I wouldn't turn down people on the basis of age if they're functionally good" – R11.

Concerns regarding age were three-fold. Firstly, age was assessed as a factor in combination with, or as a surrogate for, physiological fitness. This "perceived age" was felt to indicate the likelihood of survival for AAA repair.

"I think realistically, it's highly unlikely I would offer open repair to someone who's 85, or over, you know. Yeah, almost regardless but not completely… it's age really being a surrogate marker for general fitness," – R5

A second concern regarding age, was the concept of longevity. This influenced assessment whether the surgical insult of repair, balanced against the likelihood of rupture within the patient's lifetime, was likely to benefit the patient overall.

"So you've got to know your patient is going to benefit from that. And that means they've got to have some years to live." – R7…

"So it's not how old you are. It's how long you're going to live" – R4.

Thirdly, for those likely to benefit from repair, further consideration, relating to the durability of repair was made. A lower risk, less durable procedure (e.g., EVAR) may be sufficient for an older patient, while a younger patient might benefit from a higher risk procedure providing a more definitive solution (e.g., open repair).

"you have a 50 year old and you put an endograft in and there's a 10% intervention likelihood per annum or complication rate per annum then that's not going to last them for the rest of their life." – R9

Patient Fitness

Fitness, indicating the ability of the patient to withstand the insult of operative repair was repeatedly cited to be one of the most important factors to consider for AAA repair. This sub-theme could be broadly categorised into performance status, results of quantitative testing, psychological fitness, and frailty.

All surgeon's interviewed consistently described utilising ***lifestyle*** questions to gauge an idea of the patient's quality of life and overall day-to-day activity levels.

"So, I go a lot on their own exercise tolerance and what they do day to day. Do they do the garden? Can they walk the dog? Can they go upstairs? Are they the person that goes shopping and carries the bags? Just to get an idea of how they actually are managing, or are

they a person who is sitting at home with everything done for them, and they rarely go out?" – R7

The majority of surgeons also referenced ***walking stairs*** with the patient in clinic to assess performance status.

"Normally, in the clinic, I do assess the exercise tolerance. You know, simple questions or you know, walk them up the stairs. I think that might give me some idea of how much this patient tolerates and how strong this patient is for an open repair" – R3

"Functional fitness is much more important to me than the physiological fitness. Can they do two flights of stairs? Can they go for a long walk? What's their lifestyle like?" – R11

Although surgeon's alluded to ***quantitative tests***, such as echo or lung function, this was not often felt to be used at initial assessment, but rather as a secondary pre-operative work up, to verify suitability for AAA repair. Moreover, use of quantitative testing of physiological fitness were not uniformly performed or described. Of the eleven interviewed, only one surgeon placed significant value on the use of cardiopulmonary exercise testing to guide choice of repair.

"You develop a sixth sense about who will be able to withstand the rigours of an open operation, and so, as I said if they can walk up to a two flights of stairs without getting breathless, nearly always 99.9% of the time the echo and lung function tests are ok." - R9

"There are more objective tests than that. We might use them if the patients have some comorbidities but someone who is relatively fit and relatively young. I think we're quite happy without those tests." – R3

Psychological fitness, motivation and resilience were also expressed by some surgeon's to be important in determining the course of a patient's recovery from an AAA repair.

"The decision to treat has to be based on the patient's function and ambition." – R11

"…it gives you an idea of their mindset. And have they got the will to get over procedure and get better." – R7

Frailty is defined as an increased vulnerability from aging-associated decline across multiple systems, compromising the ability to withstand acute stressors or the everyday[37]. Although ***frailty*** was mentioned as an adjective in passing by several surgeons, only two explicitly mentioned definitions or use of formal assessment.

"I use their clinical frailty scores. And I have a fairly significant discussion on kind of their day to day activity, how independent they are, what their functional status is, what their Mets are." – R10

"So sometimes there's just a slight feeling of uneasiness about phenotype of the patient and how well they're going to cope with critical care stays and major surgery. But it's if it's not borne out in the frailty data, I go with the objective measures, not the clinical concern." – R1

Comorbid Status

Co-morbidities were generally considered both as factors contributing to the overall assessment of fitness for a general anaesthetic and surgical insult, and as indicators of patient longevity and the utility of AAA repair.

"I think co-morbidities that's a very sort of broad category…and the performance status and the co-morbidities obviously inform each other, but I think that will give you a pretty good overview of what the patient is like." – R5

The most cited co-morbidities of interest were cardiac, respiratory, and renal disease.

"I certainly am going to look at COPD, CHF, the severity of those two conditions…I'd say those two are the top." – R10

"COPD, ischaemic heart disease, renal function, those are the main comorbidities I would look for." – R3

Coronary disease was not necessarily thought to be a deterrent for surgery but may shift preference towards endovascular repair.

"…it's not a game changer. You take note of it, but it's actually a positive thing when they have had their coronaries corrected." – R7

Similarly, respiratory impairment was recognised as an important deterrent for use of general anaesthetic, shifting preference towards locoregional anaesthesia and endovascular repair.

"So probably one of the first things potentially that would make an endovascular approach under a regional anaesthetic doable and open approach unsuitable would be a respiratory issue. Because the big thing about that is you could probably do it under some form of epidural or local anaesthetic." – R7

Although the risk of renal injury was also felt to be greater for endovascular treatment, it did not generally preclude selection for AAA repair.

"...the patient would be advised that it might actually go under haemodialysis or other than that we will tell him that it's a very minimum risk. We will still operate on them." – R2

Patient Perspectives/Shared Decision Making

All surgeon's voiced support for exploring patient perspectives and for the process of shared decision making, within the realms of reasonable clinical practice.

"I mean, it's shared decision making…as long as they're reasonable candidates for both if a patient has a strong preference, I will abide by it." – R10

“I can't assume that my values as an individual are gonna be the same as his values.” – R8

Patient preference was felt to be particularly important for the selection of open repair or EVAR. Particularly when this conflicted with the surgeon’s preference.

“Patient opinion is very, very important because it's very, very contentious whether you would do an endovascular repair in a patient who would be anatomically suitable if they are fit for open repair.” - R4

Discordance between clinician and patient preference was often described to be centred around a balance between the desire for a definitive repair, and anxiety about the repair itself.

“So there's some patients who go with the flow… Whereas there'd be some patients who would be frankly devastated by the by the idea of having a diagnostic angiogram to see where the endoleak was coming from…” - R4

“They would much rather have an EVAR because they're desperately worried about the day of surgery, their mortality and death from surgery. Whereas others are kind of far more pragmatic and don’t want to keep coming back… So, what the patient desires is really important.” - R9

“I think patient anxiety does play a role. I think patient anxiety could twist your arm to one way or the other.” R6

However, it was largely felt that when patients were provided with explanation and therefore adequately informed, discordance in clinician-patient preference was most often resolved or could be accepted.

"they still need someone to sit with them who has a knowledge and experience and take them through step by step, what will happen, where's the evidence, where we stand…that relieves their anxiety and eventually they would often go for whatever you're basically offering them. So, in the end, I think as a doctor you still will lead their decision." - R6

"I think it is a time bomb to them, I do spend time trying to educate them, you know, as much as I can…. then I give them the options, although most often they ask for my recommendation and I do tend to them let them know what I think might be better, but I do leave it to them to decide." R3

"It's ultimately his [decision] regardless of what I think…As long as I'm confident that he understands the advantages and disadvantages of each option and he's made an informed choice, then I will support that." - R8

It was also described that the patient's decision making can be manipulated by use of selective emphasis during the surgeon's explanation of the procedure.

"But most of the patients will follow even if they have extensive ideas and anxiety about anything, it is just the way you present it. If obviously you are an endo man, you just want to do endo and you don't believe in open repair, you could justify that and the patient will accept." – R6

AAA Size

All surgeon's cited the aneurysm size and reaching threshold as a preliminary step in deciding whether to consider offering AAA repair.

"The first thing would be the size. Whether it has reached the threshold which is 55-millimetre maximum diameter." – R3

However, this threshold is not absolute. Rather adjustment to the aneurysm diameter threshold is used for higher risk cases, to match peri-procedural risk with rupture risk.

"I think it's because it's about matching, perioperative or peri-procedural risk to rupture risk. And so if somebody is have high peri-procedural risk…then they are maybe safer being left because their rupture risk is lower than their peri-procedural risk….So, if somebody is high, high technical risk…if they get up to 6cm then their rupture risk is higher and matching the periprocedural risks, and then the risk benefit ratio changes, doesn't it? So for me, that makes sense." - R8

While it was recognised that anatomical suitability for EVAR may reduce with increasing aneurysm size, size itself was not felt to be a determining factor regarding decision for open repair or EVAR.

"…the bigger the aneurysm the less likely it's going to be to be endovascularly repairable... I think the neck shortens as they as they expand." – R11

"aortic diameter doesn't really play a huge part in my decision as to whether to go for open or endo" – R5

Anatomical Suitability

Anatomical features were felt to be of primary importance by all surgeons. Of these, characteristics of the neck were widely felt to be the main determinant for suitability, with regards to the adequacy of the seal zone for EVAR or possible clamp placement for open repair.

"The top end is the most important anatomical factor." - R11

Thrombus burden was also cited to be a deterrent for EVAR, largely due to lack of an adequate seal zone and as a general marker of disease severity.

"If there's thrombus burden or calcification in your seal zone, I don't think that's a reasonable seal to do an EVAR on." - R10

The second most dominant anatomical feature was access, determining device deliverability and therefore the feasibility of EVAR. However, it was widely recognised that access issues could now, in some cases, be addressed using adjunctive procedures.

"So, there's no point in having a beautiful neck and nice and straight, parallel no thrombus, not angulated but then you've got no access to get into. So of course, you've got to think about the access." – R4

"I think the needs are now less, more low-profile devices, more experience with crack and pave, or angioplasty and pave." – R7

Considerable emphasis was placed on the overall anatomical constraints defined under device specific IFU (instructions for use). This was largely in recognition of the increasing body of evidence regarding superior longevity of repair and clinical outcomes in those treated within IFU, compared to those treated outside of IFU guidance.

"I think now we do accept to the importance of IFU. So, if you're outside IFU for a conventional off the shelf infrarenal stent you've really got to think hard about should you be doing that." – R7

"And so only when you stick to IFU do you get the performance that you expect….And so I guess ultimately is that an endograft that will fix the aneurysm within IFU." – R4

"If you've got a good anatomy for an EVAR it is a durable procedure. The people when we talk about when EVAR doesn't work is the ones when we go out of IFU we put in stents right, left and centre without looking into the anatomy, just for the sake of putting them in and then the results are bad." – R6

"And we know that that, you know, cases whose morphology is on IFU are likely to do better than those who are off so yeah I'd say it's a really good surrogate marker." – R5

In addition, for open repair, emphasis was placed on whether the patient had previous abdominal surgery, or had a greater body mass index, as these factors were recognised to make operative repair more difficult.

"And whether they have an hostile abdomen so, even with poor anatomy, you've got someone who's had radiotherapy and a stoma then you're still going to try and use an endograft." – R9

"…the things I think about when I'm doing open repair are, is the patient massive, because when the patient is massive, it is difficult. It's a different operation." - R4

Multidisciplinary Team Assessment

While anaesthetic opinion was held in high regard, minimal emphasis was placed on multidisciplinary meetings and multidisciplinary team assessment in guiding surgeon's opinion regarding treatment of infrarenal AAA. Moreover, multidisciplinary meetings were not routinely used for infrarenal EVAR outside of the United Kingdom.

"And but ultimately the end of the cardiopulmonary exercise test report, a consultant anaesthetist has done that test and they will put at the bottom of it this is a high risk for open or standard risk for open or EVAR… and that will… if they think they're fit, then I would obviously think they're fit" - R8

"No there's no meeting, I do it independently." – R10

Few surgeons referred to, or felt the need for, a radiologist's opinion.

"MDT-wise because we do some of the EVARs ourselves we might not consult all the time with the radiologists." – R3

Variation in attributes and relative importance, according to patient sex

Vascular surgeon's perspectives on sex-specific differences which might affect selection for AAA repair were heterogeneous. A thematic map outlining sex-specific differences in factors affecting selection for AAA repair is illustrated in Figure S3.

Some surgeons felt that there was no appreciable difference in the attributes or treatment of men and women in their clinical practice.

"I think that the risk factors are similar. I haven't seen a dramatic difference in terms of anatomy." – R11

Despite evidence regarding surgical selection rates and outcomes differing for men and women, most interviewed felt that they themselves provided **equitable care.** They were not aware of any differences in their practice or of a need to treat men and women differently.

"I treat them fairly similar." – R3

"I don't think that's actually different from what I would do in the men. No, I guess I think it would be pretty similar." – R5

Most demonstrated an appreciation of increased risk for repair amongst women, acknowledging that increased age and smaller vasculature could lead to greater issues with endovascular access. Inconsistent views were also expressed regarding the appropriate treatment size for men and women.

"We would tend to operate on smaller aneurysms for women. Now we operate on them at 50 when we just operate on men at 55. Usually, they will have hostile access for endovascular surgery." – R2

"And of course, women have probably got smaller access than men. So that may push some women out of the endovascular category that you may otherwise would have chosen an endovascular repair for" – R4

Women were also often perceived to be less fit for surgery.

"The girls are often, the ones that I've treated are probably quite a lot smaller. Skinny even. And that in itself makes you worry about frailty… So I get slightly more twitchy about women with aneurysms actually." – R1

"I think probably think scientifically their physiological reserve to undergo any major surgery is slightly different to the men. They take longer to recover. The risk of post operative complications, especially the lungs, the heart is slightly more common compared to men." – R6

Overall Ranking of Attributes

Overall, surgeon's opinions regarding the most important attributes for consideration were consistent and encompassed various measures of fitness, anatomical suitability, and patient age/longevity. Following on from these, it was felt by various surgeons that a fourth factor, patient preference, should be accounted for.

"Number one, fitness, number two age, number three aortic anatomy…the fourth factor is it depends on patient preference, because some people are very risk averse" - R9

"In general, its fitness and anatomical suitability." - R7

"I think the morphology of the aneurysm will determine what you can and can't do, and the fitness of the patient, and obviously the patients wishes. Three things." – R8

"So anatomy, I would say is first and foremost. And then their co-morbidities and clinical frailty." - R10

"The three factors should be anatomy, Life expectancy and then function…"- R3

Choice Set & Attribute Level Selection

A panel of vascular surgeons were first presented with results of the thematic analysis for both surgical selection and sex-specific differences, the scoping review of surgical risk prediction and Instructions for Use (IFU) criteria. The panel were then asked to prioritise themes for inclusion in the DCE. An iterative discussion was conducted until consensus (defined as 75% agreement) was reached regarding selection of 6 broad themes[38]. The final number of attributes were restricted to 6, considering previous work by de Bekker-Grob et al. 2012, who recommend an average 2-8 attributes – mode of 6 – which should be subdivided into clinically relevant levels (e.g., continuous (trade-off values) or categorical values)[39]. Each attribute was then discussed regarding appropriate descriptive format and levels, utilising results from the systematic review of the literature and Instructions for Use (IFU) criteria to further inform expert opinion. Again, an iterative approach was used until consensus (defined as 75% agreement) was reached[38]. See Figure S4.

Fractional Factorial Design of DCE

For this study, a full factorial design was too large to be feasible (i.e., 2 attributes with 2 levels and 4 attributes with 4 levels would lead to $2^2 \times 4^4 = 1024$ scenarios). Therefore, before pilot testing a fractional factorial design, (a subset of all possible combinations,) was constructed to using an Orthogonal Main Effects Plan (OMEP) design to ensure orthogonality, (minimal correlation between different attribute levels), attribute level balance and minimal overlap, assuming independence of two-factor interactions (Experimental Plan Code Number 87)[40].

Survey Construction

The survey was constructed in 3 parts using Qualtrics XM Platform™ (Qualtrics, Provo, UT), an online self-administration survey platform. First, an introductory section was constructed to explain the subject of the study, who is carrying out the survey and how the results will be used. To avoid bias, participants were be blinded to the primary objective of the study. A participant information sheet was provided, and the user was required at this point to

confirm consent for their data to be used in the study. The second section contained the 16 discrete choice scenarios, as well as an additional scenario with clear dominance to assess choice desirability, and a repeated scenario to assess choice consistency. The order of case scenarios was randomised, using the Qualtrics platform, to minimise ordering effects[41]. The final section requested information regarding pre-selected factors which may contribute to preference heterogeneity, as indicated by previous publications, namely, surgeon sex, years of clinical experience, the percentage of AAA repairs performed as EVAR, and the country in which the surgeon clinically practices[42–45].

Pilot Testing

Pilot testing was conducted by to determine whether the volume of choice tasks was appropriate (e.g., prevented user fatigue), to assess the respondent efficiency, and to assess the perceived realistic nature of the design (e.g., suitable attribute levels and unambiguous descriptors)[46]. A talk out loud approach was used to enable for dynamic feedback and an iterative approach was used to modify the questionnaire in accordance with constructive feedback. The DCE survey was completed by 18 vascular surgeons and was generally well received. 18 case scenarios were felt to be a manageable workload by most surgeons. Emphasis was made that scenarios should be answered prior to demographic questions to prevent disengagement or confusion in the questionnaire format. Request was made to extend the age range from 55-85 to 52-88 to encompass more of the AAA population. Frailty as an attribute was variably received, but generally felt to be confusing as it did not necessarily directly relate to decision for AAA repair. Use a of symbol of a wheelchair bound patient was also felt to be “leading”. Following, feedback, it was decided that this attribute should be changed to 4 levels of anaesthetic risk, (low, moderate, high and very high,) which encompassed medical fitness and the likelihood of immediately surviving the AAA repair. As the number of attributes and levels remained consistent following pilot testing, the selected fractional factorial design was retained for the final DCE. (See Table S1.)

Power Calculation

A sample size calculation was performed using a formula established by Orme *et al.* for assessment of main effects, assuming a maximum of 4 attribute levels, 8 choice tasks and a binary choice design[47].

L_k - levels, k – attributes, J = alternatives, T = choice tasks, N = minimum number

N = 500 x max (L_k)/J*T

= 500*4/2*16

= 63 respondents completing 16 choice sets, or 125 respondents completing 8 choice sets.

As a preliminary assessment of feasibility, it was calculated that a 15 percent respondent rate from registered consultant vascular surgeons in the UK (78/522) completing 16 scenarios would provide ~1248 scenarios for analysis.

Following pilot testing, parameter estimates were then used to assess the minimum sample size requirements which accounted for the statistical power of hypothesis tests on the estimated co-efficient, as per methodology previously published by de-Bekker Grob[1]. For the primary hypothesis, it was calculated that a minimum of 9 respondents completing 16 choice scenarios would provide a statistical power of 0.8 (assuming an $\alpha = 0.05$) to assess whether patient sex has a significant effect on selection for AAA repair, while a minimum of 140 respondents completing 16 choice scenarios with a statistical power of 0.8 (assuming an $\alpha = 0.05$) would be needed to assess the effect of patient sex on selection for EVAR. Therefore assuming 20% attrition rate, a target recruitment value of 168 respondents was chosen.

Administration of DCE Survey

Participants were eligible for inclusion if they were a consultant or senior specialist registrar vascular surgeon with experience of both open and endovascular aortic repair. The survey was conducted in English and so those with a significant language barrier were not eligible

for inclusion. Pre-specified withdrawal criteria included an incomplete survey response, (e.g., failure to consent or <50% of questions answered), and failure to demonstrate clinical equipoise (e.g., all questions answered as one alternative).

As the DCE required widespread participation of consultant vascular surgeons with experience in vascular surgery, a multifaceted approach was utilised, including in person recruitment at international conferences (e.g., Charing Cross Symposium, British Society for Endovascular Therapy, and the Women's Vascular Summit, USA), distribution via Twitter (e.g., Imperial Vascular account) and via dedicated collaborative research networks such as the VQI (Vascular Quality Initiative, USA) and VASCUNET. To avoid introduction of bias the hypothesis guiding development of the DCE was blinded from the participants. (See Supplemental Materials Questionnaire.)

Statistical Analysis

Econometric Analysis

Descriptive statistics were compared using standard parametric and non-parametric testing as appropriate. Continuous data was centred and scaled, utilising the median value for age (70 years) and the threshold value (55mm) for AAA size. All analyses were conducted in RStudio, using the Apollo R package[48,49].

Choice data were first analysed using logistic regression models with robust variance estimates for analysis A (selection for repair) and analysis B (selection for EVAR). Second, an error component model was used to relax the IIA (irrelevance of independent alternatives assumption) by classifying alternative specific constants as random (assuming a normal distribution,) and assuming preferences for scenario attributes remained constant in the sample. Following this, interaction between patient sex and further attributes were individually introduced and assessed for each model. To assess for preference heterogeneity associated with surgeon characteristics, pre-specified subgroup analyses were conducted for surgeon gender (men or women), level of experience (early: <10 years, mid-level: 10-20 years or experienced: >20 years), preference for EVAR repair (categorised into 1st "low",

2/3rd "mid-range" and 4th "high" quartiles) and geographical location (categorised as UK, Europe, North America or other).

Parameter estimates were presented with robust (Huber-White) standard errors (accounting for heteroskedasticity), t-values and a 2-sided p value for which a value of <0.05 was considered statistically significant. Models were compared using likelihood ratio chi-square statistic and Akaike Information Criterion and goodness of fit was estimated using adjusted pseudo R^2. Assessment of model performance was conducted using share prediction tests - the difference between times predicted and times chosen and fits tests, which given the estimates of a model, it compares the log-likelihood at the observation level across categories of observations.

Marginal rates of substitution were calculated with AAA size (mm) as the denominator and utilising the delta method for standard error estimation, to enable comparison of relative importance between attributes and subgroup models[50]. To convey results in a manner which is more meaningful for vascular surgeons, the mean percentage difference in choice probabilities for women and men were conducted for a variety of AAA patient scenarios.

Utility Function

The utility function was defined as follows:

Analysis 1:

$V_{surgical\_repair} = ASC_0 + ß_1 female\_sex + ß_2 age + ß_3 anaes\_mod + ß_4 anaes\_high + ß_5 anaes\_vhigh + ß_6 patient\_anxiety + ß_7 AAA\_diameter + ß_8 anatomy\_neck + ß_9 anatomy\_access + ß_{10} anatomy\_neck{+}access$

$V_{turndown} = ASC_1$

Analysis 2:

$V_{endovascular\_repair}$ = ASC_0 + $ß_1$female_sex + $ß_2$age + $ß_3$anaes_mod + $ß_4$anaes_high + $ß_5$anaes_vhigh + $ß_6$patient_anxiety + $ß_7$AAA_diameter + $ß_8$anatomy_neck + $ß_9$anatomy_access + $ß_{10}$anatomy_neck + access

V_{open_repair} = ASC_1

Where:

V - represents the systematic utility for the selected invention.

ASC_0 - represents the alternative specific constant, which captures the variation in choice not explained by the attributes, which, for this analysis is set as a constant to 0.

ASC_1 - represents the alternative specific constant, which captures the variation in choice not explained by the attributes, for the reference level and at the means and dummy bases. The reference level is "turndown" for analysis 1 and "open repair" for analysis 2. For the error component model, this is set as a random parameter, assuming a normal distribution, and using 500 mhls (Modified Latin Hypercube Sampling) inter-individual draws[51].

$ß_{1\text{-}10}$ - represent specific parameter weights for coefficients associated with the attribute levels specified. A positive value indicates that the attribute level combination was preferred relative to the reference level. A larger absolute value indicates a stronger preference.

ε – stochastic disturbance term representing unobserved characteristics.

Assessment of Consistency and Validity

Quality of choice data was assessed using the following criteria:

1. *Choice desirability*:
   A scenario with all patient-related features set to their best (i.e., most attractive for AAA repair). In this case, the respondent failed the desirability test if they did offer an AAA repair.
2. *Choice stability*:

Scenario 5 was repeated within the choice set to assess for consistency in surgeon's choice for AAA repair and for EVAR.

3. *Clinical Equipoise:*
   Analyses were conducted to examine whether a participant selected one choice (e.g., Q1 "No" or for Q2 "Yes/EVAR") for >90% of questions, as this may represent significant clinician choice bias. For these respondents, the survey response time, compared to other respondents, was scrutinised to assess the level of engagement. A shorter than average response time was pre-specified to indicate poor engagement, with subsequent exclusion of these data from analyses.

Sensitivity Analysis

Sensitivity analyses were used to explore the effect of forced choice on attribute effects for analysis B, by using a subgroup of cases for whom "repair" was selected in question A. Stability of findings was assessed using subgroup analyses following exclusion of choice sets for respondents who failed the assessments of consistency and validity (e.g., choice desirability, choice stability and clinical equipoise tests).

Supplemental Figures and Tables

Figure S1.

Schematic representation of discrete choice experiment development process.

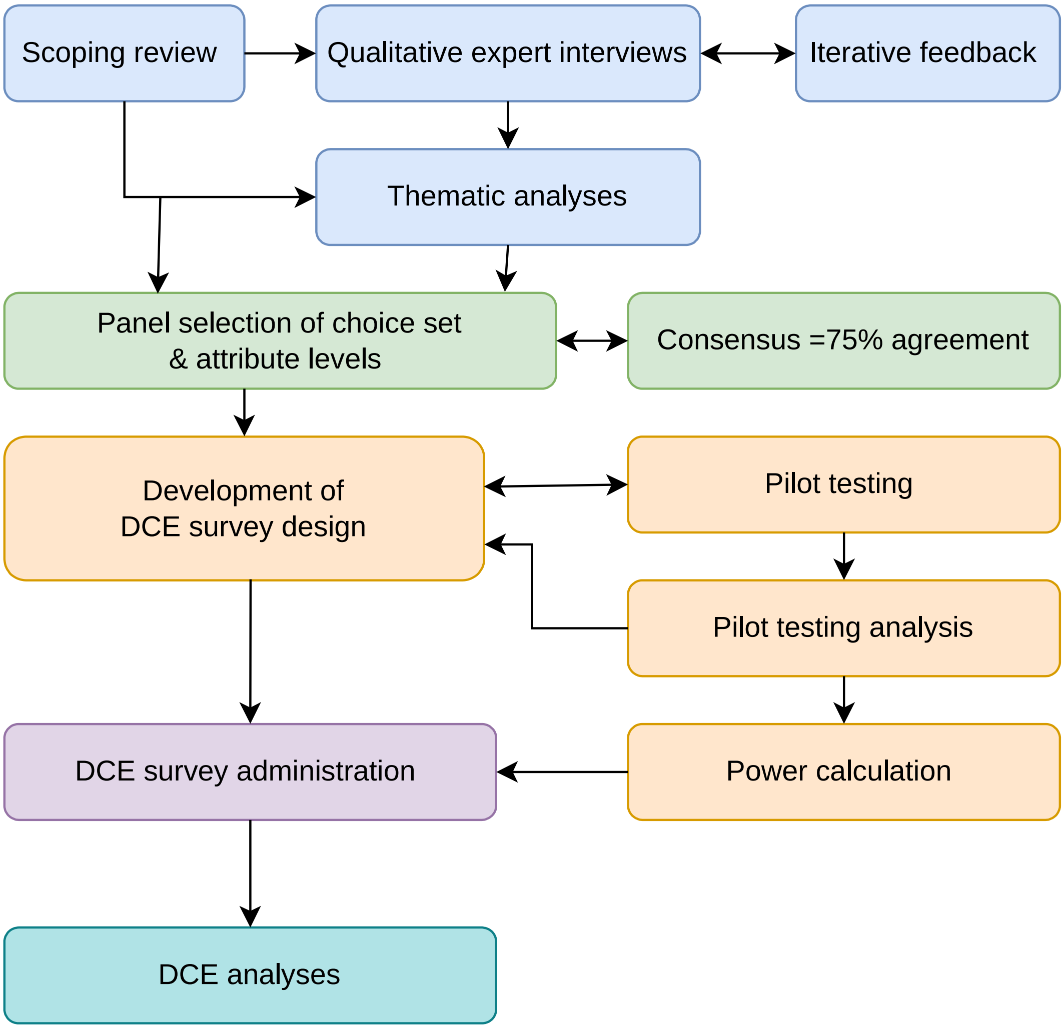

Figure S2.

Thematic map outlining factors affecting selection for AAA repair. Black dashed lines represent a relationship between subthemes or categories, red dashed lines represent conflict between subthemes or categories. Abbreviations: AAA – abdominal aortic aneurysm, BMI – body mass index, EVAR – endovascular aortic repair, IFU – instructions for use categories, PAD – peripheral arterial disease, QoL – quality of life.

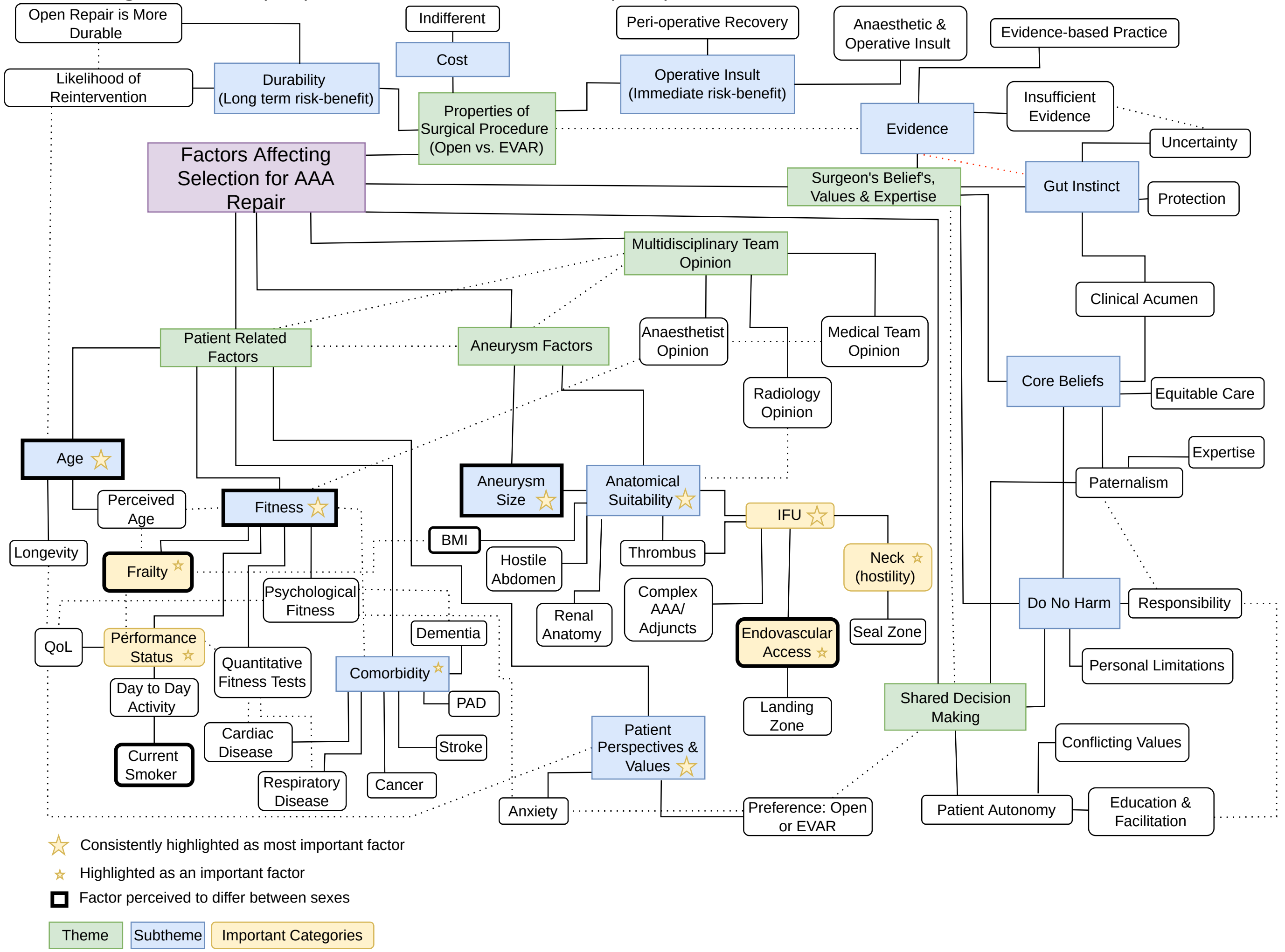

Figure S3.

Thematic map outlining sex-specific differences in factors affecting selection for AAA repair. Black dashed lines represent a relationship between subthemes or categories, red dashed lines represent conflict between subthemes or categories.

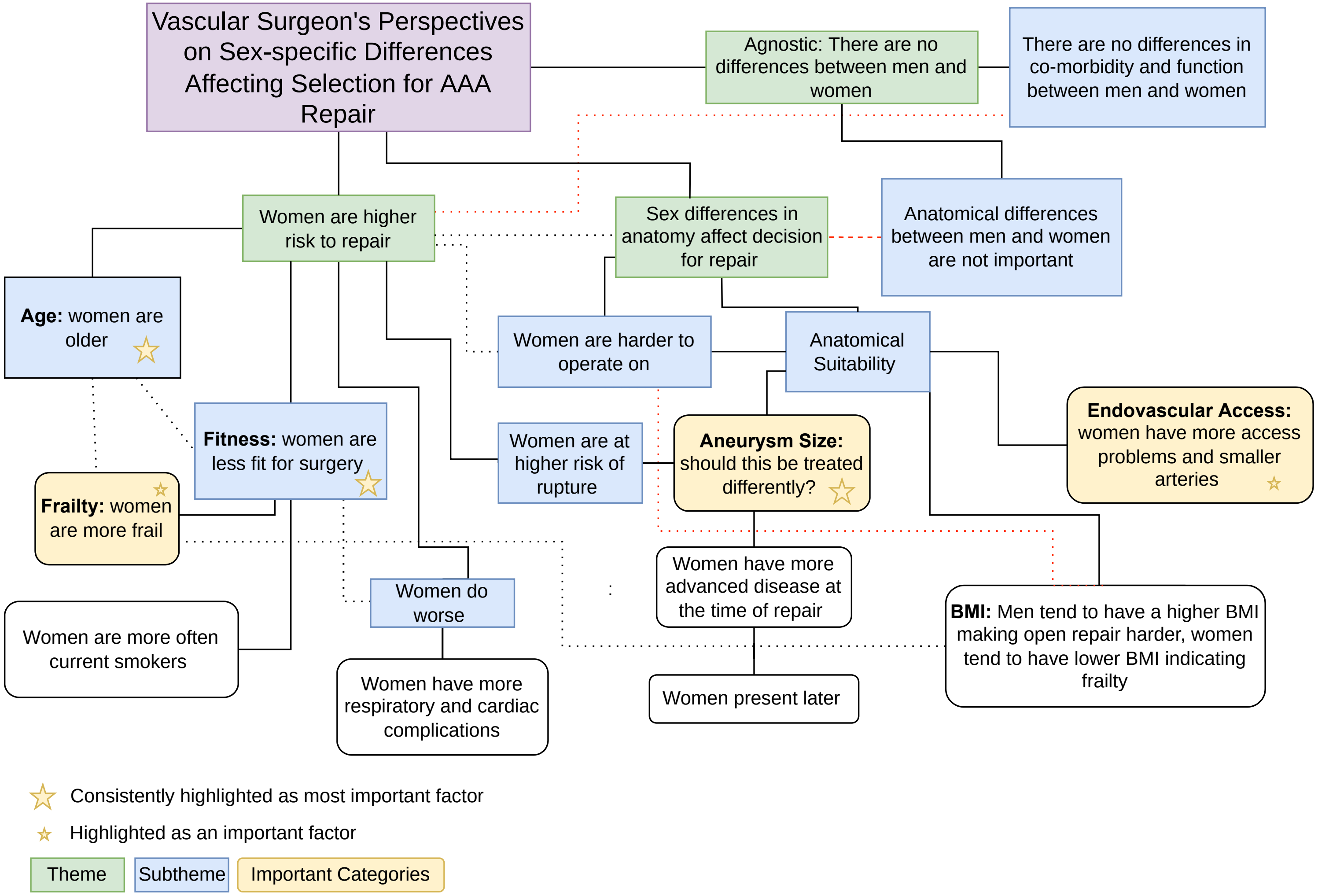

Figure S4.

Narrowed down thematic map outlining the most important factors affecting selection for AAA repair. Black dashed lines represent a relationship between subthemes or categories. Abbreviations: AAA – abdominal aortic aneurysm, BMI – body mass index, EVAR – endovascular aortic repair, IFU – instructions for use categories, PAD – peripheral arterial disease, QoL – quality of life.

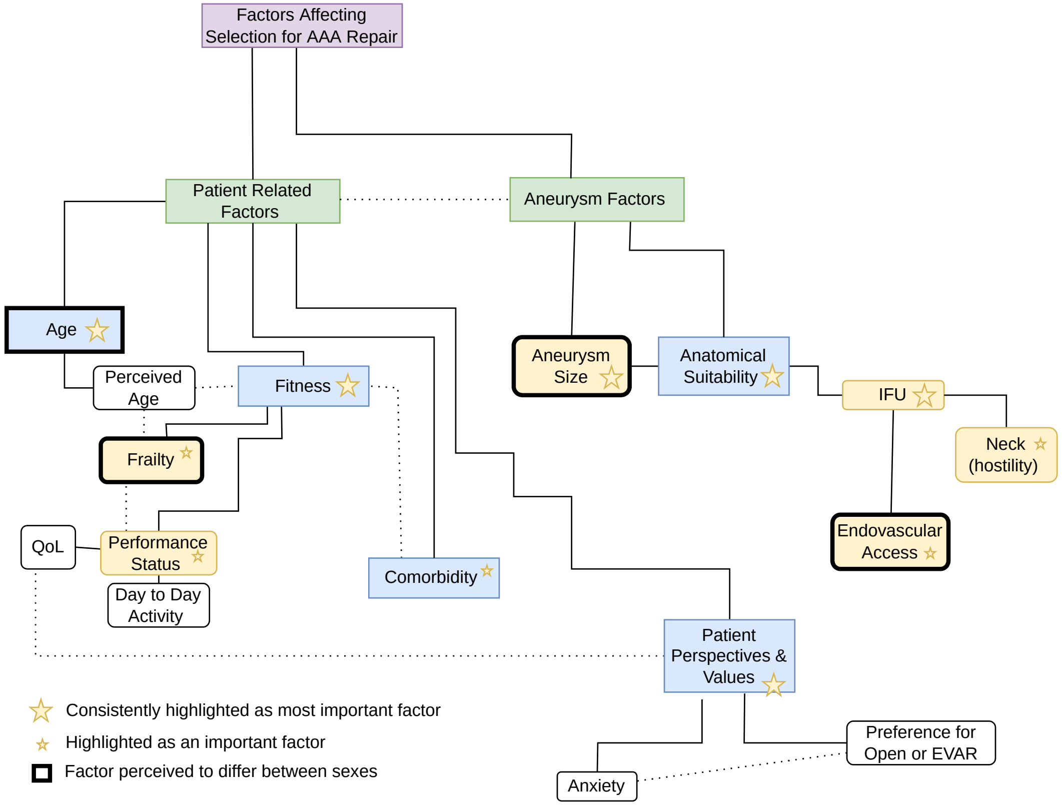

Table S1. Pilot study and Final DCE Attributes and Levels.

Pilot Attribute	Name	Levels	Interpretations of Co-efficient	Final Attribute	Name	Levels	Interpretation of Co-efficient
Patient sex	sex0	Man (reference level)	Indirect utility of being female compared to male	Patient sex	sex0	Man(reference level)	Indirect utility of being female compared to male
	sex1	Woman			sex1	Woman	
Age (years)	age	55, 65, 75, 85	Indirect utility of 1 year increase in recipient age	Age (years)	age	52, 64, 76, 88	Indirect utility of 1 year increase in recipient age
Frailty	cfs0	Managing well/Easily climbs two flights of stairs	Indirect utility of mild, moderate and severe frailty compared to managing well	Anaesthetic Risk	anaes0	Anaesthetic risk = Low	Indirect utility of moderate, high and very high anaesthetic risk compared to managing well
	cfs1	Mildly frail /Breathless climbing two flights of stairs			anaes1	Anaesthetic risk = Moderate	
	cfs2	Moderately frail/Slow & breathless climbing two flights of stairs			anaes2	Anaesthetic risk = High	
	cfs3	Severely frail /Cannot climb two flights of stairs			anaes3	Anaesthetic risk = Very High	
Patient perspectives	per0	The patient is happy to follow your recommendation (reference level)	Indirect utility of patient opinion (anxiety regarding surgery) rather than no opinion	Patient perspective	anxiety0	The patient wants to have their aneurysm repaired (reference level)	Indirect utility of patient opinion (anxiety regarding surgery) rather than no opinion
	per1	The patient is anxious about the risks of surgery.			anxiety1	The patient is worried about the risks of surgery	
AAA size (mm)	diameter	50, 55, 60, 65	Indirect utility of 1mm increase in AAA diameter	AAA size (mm)	diameter	50, 55, 60, 65	Indirect utility of 1mm increase in AAA diameter
Anatomy	anat0	Repair is on IFU (reference level)	Indirect utility of hostile neck, hostile access or hostile neck and access compared to anatomy on IFU	Anatomy	anat0	Repair is on IFU (reference level)	Indirect utility of hostile neck, hostile access or hostile neck and access compared to anatomy on IFU
	anat1	Hostile neck requiring adjuncts or complex repair			anat1	Hostile neck will require adjuncts or complex repair	
	anat2	Hostile access will require an adjunctive procedure			anat2	Hostile access will require an adjunctive procedure	
	anat3	Hostile neck requiring adjuncts or complex repair + Hostile access will require an adjunctive procedure			anat3	Hostile neck will require adjuncts or complex repair + Hostile access will require an adjunctive procedure	

Table S2. Summary of respondent characteristics.

Characteristic	Number of Respondents, n	%
**Gender**		
Man	132	72.53
Woman	42	23.08
Non-Binary	1	0.55
Missing	7	3.85
**Years of Surgical Experience**		
<10 years	70	38.46
10-20 years	69	37.91
>20 years	36	19.78
Missing	7	3.85
**Percentage EVAR**		
Median (IQR)	70 (40-100)	
**Location of Clinical Practice**		
Europe	48	26.37
Latin America	6	3.30
Middle East	9	4.95
North America	45	24.73
South Africa	1	0.55
Southeast Asia	4	2.20
UK	60	32.97
Missing	9	4.95

Table S3. Surgeon gender subgroups: AAA repair versus no repair.

Surgeon Gender	Men (n = 132)			Women (n = 42)		
Attribute MRS	Estimate	Lower 95% CI	Upper 95% CI	Estimate	Lower 95% CI	Upper 95% CI
**Woman**	3.92	2.90	4.94	3.62	1.38	5.87
**Age**	-0.19	-0.23	-0.14	-0.17	-0.23	-0.12
**Anaesthetic risk - moderate**	0.75	-0.10	1.60	-0.37	-1.90	1.15
**Anaesthetic risk - high**	0.34	-1.00	1.69	0.86	-1.40	3.13
**Anaesthetic risk - very high**	-4.36	-5.87	-2.84	-4.10	-7.13	-1.08
**Hostile neck anatomy**	0.57	-0.41	1.55	1.54	-0.24	3.32
**Hostile access anatomy**	2.78	1.79	3.76	2.91	1.58	4.25
**Hostile neck and access anatomy**	-3.49	-4.91	-2.06	-3.22	-6.25	-0.19
**Patient reported anxiety**	0.76	-0.04	1.56	0.00	-1.04	1.04

Abbreviations: CI – confidence interval, MRS – Marginal Rate of Substitution (*denominator AAA size (mm)). Attribute reference levels: Man, Anaesthetic risk – low, Anatomy on instructions for use (IFU), Patient wants procedure.

Table S4. Surgeon gender subgroups: EVAR versus open repair

Surgeon Gender	Men (n = 132)			Women (n = 42)		
Attribute MRS	Estimate	Lower 95% CI	Upper 95% CI	Estimate	Lower 95% CI	Upper 95% CI
Woman	2.05	0.75	3.35	4.88	1.04	8.72
Age	0.39	0.28	0.50	0.60	0.26	0.93
Anaesthetic risk - moderate	3.80	1.91	5.70	1.43	-2.50	5.35
Anaesthetic risk - high	14.35	10.63	18.08	19.26	9.68	28.85
Anaesthetic risk - very high	15.47	11.79	19.14	17.56	7.70	27.42
Hostile neck anatomy	-2.89	-5.01	-0.76	1.45	-3.00	5.90
Hostile access anatomy	-10.21	-12.98	-7.44	-11.60	-18.45	-4.75
Hostile neck and access anatomy	-9.33	-12.18	-6.47	-8.97	-15.42	-2.52
Patient reported anxiety	-1.94	-3.36	-0.51	-1.67	-4.95	1.61

*Abbreviations: CI – confidence interval, MRS – Marginal Rate of Substitution (*denominator AAA size (mm)).* Attribute reference levels: Man, Anaesthetic risk – low, Anatomy on instructions for use (IFU), Patient wants procedure.

Table S5. Surgeon experience level subgroups: AAA repair versus no repair.

Surgeon Experience	Early (<10 years (n = 70))			Mid-level (10-20 years (n = 69))			Experienced (>20 years (n = 36))		
**Attribute MRS**	**Estimate**	**Lower 95% CI**	**Upper 95% CI**	**Estimate**	**Lower 95% CI**	**Upper 95% CI**	**Estimate**	**Lower 95% CI**	**Upper 95% CI**
**Woman**	3.48	1.86	5.11	4.57	3.14	5.99	3.20	0.86	5.53
**Age**	-0.14	-0.21	-0.07	-0.21	-0.27	-0.16	-0.23	-0.31	-0.15
**Anaesthetic risk - moderate**	0.91	-0.29	2.11	-0.17	-1.17	0.84	0.51	-1.38	2.40
**Anaesthetic risk - high**	1.93	-0.09	3.95	-0.92	-2.61	0.76	-0.17	-2.60	2.26
**Anaesthetic risk - very high**	-2.94	-5.13	-0.74	-5.57	-7.73	-3.41	-4.72	-7.75	-1.70
**Hostile neck anatomy**	1.85	0.53	3.18	0.32	-1.10	1.74	0.11	-2.06	2.28
**Hostile access anatomy**	2.31	0.83	3.80	3.18	2.09	4.28	2.85	1.02	4.68
**Hostile neck and access anatomy**	-2.30	-4.04	-0.57	-4.29	-6.51	-2.06	-3.57	-6.82	-0.32
**Patient reported anxiety**	0.44	-0.63	1.51	0.13	-0.91	1.17	1.66	0.12	3.21

*Abbreviations: CI – confidence interval, MRS – Marginal Rate of Substitution (*denominator AAA size (mm)).* Attribute reference levels: Man, Anaesthetic risk – low, Anatomy on instructions for use (IFU), Patient wants procedure.

Table S6. Surgeon experience level subgroups: EVAR versus open repair

**Surgeon Experience**	**Early (<10 years (n = 70))**			**Mid-level (10-20 years (n = 69))**			**Experienced (>20 years (n = 36))**		
**Attribute MRS**	**Estimate**	**Lower 95% CI**	**Upper 95% CI**	**Estimate**	**Lower 95% CI**	**Upper 95% CI**	**Estimate**	**Lower 95% CI**	**Upper 95% CI**
**Woman**	3.25	0.37	6.13	2.89	1.22	4.56	0.66	-1.49	2.81
**Age**	0.62	0.44	0.81	0.40	0.24	0.56	0.21	0.02	0.39
**Anaesthetic risk - moderate**	1.66	-1.41	4.73	4.57	1.92	7.21	3.59	0.27	6.90
**Anaesthetic risk - high**	20.25	13.06	27.43	13.44	8.55	18.33	11.28	5.08	17.47
**Anaesthetic risk - very high**	20.82	13.52	28.12	13.21	8.84	17.59	13.36	6.65	20.08
**Hostile neck anatomy**	-3.49	-7.18	0.19	-1.52	-4.12	1.07	-1.40	-5.59	2.79
**Hostile access anatomy**	-13.19	-18.26	-8.11	-10.36	-13.94	-6.79	-7.45	-12.40	-2.51
**Hostile neck and access anatomy**	-12.47	-18.03	-6.90	-7.70	-11.21	-4.19	-8.52	-13.42	-3.62
**Patient reported anxiety**	-3.14	-6.25	-0.03	-1.21	-2.64	0.22	-0.44	-3.04	2.16

*Abbreviations: CI – confidence interval, MRS – Marginal Rate of Substitution (*denominator AAA size (mm)).* Attribute reference levels: Man, Anaesthetic risk – low, Anatomy on instructions for use (IFU), Patient wants procedure.

Table S7. Use of EVAR in clinical practice subgroups: AAA repair versus no repair.

**Use of EVAR**	**Low (1st quartile (n = 31))**			**Mid-range (2nd/3rd quartiles (n = 70))**			**High (4th quartile (n = 54))**		
**Attribute MRS**	**Estimate**	**Lower 95% CI**	**Upper 95% CI**	**Estimate**	**Lower 95% CI**	**Upper 95% CI**	**Estimate**	**Lower 95% CI**	**Upper 95% CI**
**Woman**	3.82	1.94	5.69	4.09	2.55	5.63	4.17	2.34	6.00
**Age**	-0.17	-0.25	-0.08	-0.17	-0.23	-0.12	-0.21	-0.29	-0.12
**Anaesthetic risk - moderate**	-0.05	-1.76	1.66	-0.30	-1.58	0.99	1.73	0.52	2.94
**Anaesthetic risk - high**	-1.84	-4.31	0.62	0.09	-1.73	1.92	2.03	-0.40	4.46
**Anaesthetic risk - very high**	-5.77	-8.81	-2.72	-4.02	-6.01	-2.04	-3.90	-6.58	-1.22
**Hostile neck anatomy**	-0.56	-2.39	1.27	0.90	-0.46	2.26	1.90	0.22	3.57
**Hostile access anatomy**	3.47	1.33	5.62	2.18	0.93	3.43	3.26	1.88	4.65
**Hostile neck and access anatomy**	-3.23	-6.18	-0.28	-3.25	-5.31	-1.19	-3.00	-5.39	-0.60
**Patient reported anxiety**	0.30	-1.03	1.63	0.73	-0.29	1.75	0.18	-1.21	1.58

*Abbreviations: CI – confidence interval, EVAR – endovascular aortic repair, MRS – Marginal Rate of Substitution (*denominator AAA size (mm)).*

Attribute reference levels: Man, Anaesthetic risk – low, Anatomy on instructions for use (IFU), Patient wants procedure.

Table S8. Use of EVAR in clinical practice subgroups: EVAR versus open repair.

Use of EVAR	Low (1st quartile (n = 31))			Mid-range (2nd/3rd quartiles (n = 70))			High (4th quartile (n = 54))		
**Attribute MRS**	**Estimate**	**Lower 95% CI**	**Upper 95% CI**	**Estimate**	**Lower 95% CI**	**Upper 95% CI**	**Estimate**	**Lower 95% CI**	**Upper 95% CI**
**Woman**	0.45	-2.32	3.23	3.75	1.76	5.73	2.61	-0.18	5.39
**Age**	0.37	0.14	0.60	0.43	0.28	0.59	0.54	0.28	0.79
**Anaesthetic risk - moderate**	0.92	-3.80	5.65	2.77	0.43	5.10	6.36	2.42	10.31
**Anaesthetic risk - high**	18.10	9.90	26.30	13.13	8.35	17.91	16.67	8.11	25.24
**Anaesthetic risk - very high**	19.65	10.81	28.49	15.12	10.36	19.88	15.32	7.65	22.99
**Hostile neck anatomy**	-5.46	-9.89	-1.03	-2.77	-5.43	-0.11	0.82	-4.06	5.69
**Hostile access anatomy**	-15.78	-22.52	-9.04	-11.22	-15.19	-7.25	-8.48	-13.42	-3.53
**Hostile neck and access anatomy**	-12.99	-18.36	-7.61	-10.19	-14.31	-6.07	-6.49	-12.26	-0.72
**Patient reported anxiety**	-4.79	-8.52	-1.05	-2.31	-4.32	-0.30	0.69	-2.19	3.57

*Abbreviations: CI – confidence interval, MRS – Marginal Rate of Substitution (*denominator AAA size (mm))*

Table S9. Geographical location subgroups: AAA repair versus no repair.

Geographical Location	United Kingdom (n = 60)			Europe (n = 48)			North America (n = 45)			World – Other (n = 20)		
**Attribute MRS**	**Estimate**	**Lower 95% CI**	**Upper 95% CI**	**Estimate**	**Lower 95% CI**	**Upper 95% CI**	**Estimate**	**Lower 95% CI**	**Upper 95% CI**	**Estimate**	**Lower 95% CI**	**Upper 95% CI**
**Woman**	4.90	3.50	6.30	3.94	1.98	5.90	3.18	0.72	5.65	0.16	-2.01	2.33
**Age**	-0.19	-0.26	-0.13	-0.27	-0.35	-0.18	-0.11	-0.19	-0.03	-0.23	-0.35	-0.10
**Anaesthetic risk - moderate**	1.01	-0.40	2.41	0.26	-1.31	1.82	0.09	-1.20	1.39	0.23	-1.44	1.90
**Anaesthetic risk - high**	-2.16	-4.08	-0.24	1.91	-0.48	4.31	1.53	-0.44	3.50	2.62	0.23	5.01
**Anaesthetic risk - very high**	-6.56	-8.97	-4.15	-4.75	-7.76	-1.74	-1.66	-4.07	0.74	-0.52	-3.91	2.88
**Hostile neck anatomy**	-1.99	-3.56	-0.41	1.54	-0.14	3.21	2.38	0.62	4.15	3.66	1.65	5.68
**Hostile access anatomy**	2.45	0.83	4.08	4.16	2.85	5.46	1.45	-0.07	2.97	4.19	2.35	6.04
**Hostile neck and access anatomy**	-5.77	-8.43	-3.10	-3.42	-5.88	-0.97	-1.35	-3.79	1.08	-1.04	-3.25	1.18
**Patient reported anxiety**	2.01	0.69	3.32	-0.35	-1.54	0.85	-0.08	-1.33	1.16	1.47	-0.20	3.14

*Abbreviations: CI – confidence interval, EVAR – endovascular aortic repair, MRS – Marginal Rate of Substitution (*denominator AAA size (mm)).*

Attribute reference levels: Man, Anaesthetic risk – low, Anatomy on instructions for use (IFU), Patient wants procedure.

Table S10. Geographical location subgroups: EVAR versus open repair

Geographical Location	United Kingdom (n = 60)			Europe (n = 48)			North America (n = 45)			World – Other (n = 20)		
**Attribute MRS**	**Estimate**	**Lower 95% CI**	**Upper 95% CI**	**Estimate**	**Lower 95% CI**	**Upper 95% CI**	**Estimate**	**Lower 95% CI**	**Upper 95% CI**	**Estimate**	**Lower 95% CI**	**Upper 95% CI**
**Woman**	2.87	0.67	5.06	1.56	-0.86	3.97	3.02	0.43	5.61	3.48	-0.23	7.18
**Age**	0.49	0.28	0.70	0.48	0.28	0.69	0.42	0.23	0.61	0.19	-0.03	0.40
**Anaesthetic risk - moderate**	3.06	-0.08	6.20	1.97	-1.03	4.97	4.41	1.18	7.65	5.17	-0.22	10.57
**Anaesthetic risk - high**	17.91	11.13	24.68	16.43	10.66	22.21	10.99	4.97	17.01	13.71	2.48	24.93
**Anaesthetic risk - very high**	21.17	13.59	28.75	14.14	8.28	20.00	11.85	6.31	17.39	14.32	3.52	25.12
**Hostile neck anatomy**	-4.49	-7.44	-1.53	-1.32	-4.93	2.29	0.42	-3.04	3.87	-5.01	-14.04	4.03
**Hostile access anatomy**	-13.58	-18.71	-8.45	-9.57	-14.54	-4.61	-7.59	-11.43	-3.75	-12.81	-22.94	-2.68
**Hostile neck and access anatomy**	-12.09	-16.84	-7.33	-9.15	-12.77	-5.53	-5.33	-10.77	0.12	-12.76	-23.92	-1.60
**Patient reported anxiety**	-4.24	-6.83	-1.65	-1.13	-3.55	1.29	-1.30	-3.61	1.01	1.66	-2.59	5.91

*Abbreviations: CI – confidence interval, EVAR – endovascular aortic repair, MRS – Marginal Rate of Substitution (*denominator AAA size (mm)).*

Attribute reference levels: Man, Anaesthetic risk – low, Anatomy on instructions for use (IFU), Patient wants procedure.

Table S11. Assessment of consistency & validity: AAA repair versus no repair.

Consistency & Validity	Whole Cohort ()			Choice Stability Subgroup			Choice Desirability & Equipoise Subgroup			Choice Stability, Desirability & Equipoise		
**Attribute MRS**	**Estimate**	**Lower 95% CI**	**Upper 95% CI**	**Estimate**	**Lower 95% CI**	**Upper 95% CI**	**Estimate**	**Lower 95% CI**	**Upper 95% CI**	**Estimate**	**Lower 95% CI**	**Upper 95% CI**
**Woman**	3.86	2.93	4.79	3.80	2.73	4.87	3.81	2.82	4.79	3.78	2.70	4.87
**Age**	-0.19	-0.22	-0.15	-0.20	-0.24	-0.16	-0.18	-0.22	-0.15	-0.20	-0.23	-0.16
**Anaesthetic risk - moderate**	0.44	-0.28	1.16	0.47	-0.25	1.18	0.38	-0.37	1.13	0.34	-0.40	1.09
**Anaesthetic risk - high**	0.39	-0.75	1.53	1.04	-0.15	2.24	0.51	-0.64	1.66	0.93	-0.24	2.11
**Anaesthetic risk - very high**	-4.33	-5.63	-3.04	-3.73	-5.20	-2.26	-4.33	-5.70	-2.96	-4.10	-5.61	-2.60
**Hostile neck anatomy**	0.93	0.09	1.77	1.50	0.59	2.41	0.73	-0.14	1.61	1.16	0.25	2.07
**Hostile access anatomy**	2.80	2.01	3.60	2.91	2.09	3.73	2.60	1.78	3.43	2.74	1.94	3.53
**Hostile neck and access anatomy**	-3.28	-4.56	-2.01	-2.86	-4.20	-1.53	-3.81	-5.18	-2.44	-3.33	-4.75	-1.90
**Patient reported anxiety**	0.46	-0.18	1.10	0.52	-0.14	1.18	0.42	-0.24	1.09	0.46	-0.22	1.14

*Abbreviations: CI – confidence interval, MRS – Marginal Rate of Substitution (*denominator AAA size (mm)).* Attribute reference levels: Man, Anaesthetic risk – low, Anatomy on instructions for use (IFU), Patient wants procedure.

Table S12. Assessment of consistency & validity: EVAR versus open repair.

Consistency & Validity	Whole Cohort			Choice Stability Subgroup			Choice Equipoise Subgroup			Choice Stability & Equipoise			AAA Repair Only Subgroup		
**Attribute MRS**	**Estimate**	**Lower 95% CI**	**Upper 95% CI**	**Estimate**	**Lower 95% CI**	**Upper 95% CI**	**Estimate**	**Lower 95% CI**	**Upper 95% CI**	**Estimate**	**Lower 95% CI**	**Upper 95% CI**	**Estimate**	**Lower 95% CI**	**Upper 95% CI**
**Woman**	2.57	1.30	3.84	2.38	0.97	3.79	2.36	0.91	3.80	2.09	0.48	3.69	7.26	0.90	13.63
**Age**	0.45	0.34	0.56	0.48	0.36	0.60	0.46	0.34	0.58	0.50	0.36	0.64	1.49	0.54	2.43
**Anaesthetic risk - moderate**	3.34	1.64	5.04	3.51	1.64	5.38	3.20	1.33	5.06	3.21	1.19	5.23	10.95	3.12	18.77
**Anaesthetic risk - high**	15.93	12.33	19.53	16.25	12.15	20.35	16.63	12.50	20.77	16.91	12.23	21.59	49.42	18.86	79.98
**Anaesthetic risk - very high**	16.31	12.74	19.89	16.52	12.49	20.54	17.61	13.50	21.72	17.68	13.12	22.25	50.51	16.78	84.24
**Hostile neck anatomy**	-2.19	-4.12	-0.26	-2.33	-4.61	-0.06	-2.91	-5.02	-0.80	-3.27	-5.75	-0.80	-6.15	-13.02	0.72
**Hostile access anatomy**	-10.88	-13.51	-8.26	-11.14	-14.03	-8.25	-11.82	-14.87	-8.76	-12.34	-15.71	-8.98	-23.67	-36.17	-11.17
**Hostile neck and access anatomy**	-9.79	-12.43	-7.14	-9.53	-12.49	-6.56	-10.34	-13.29	-7.38	-9.98	-13.29	-6.68	-25.33	-40.82	-9.85
**Patient reported anxiety**	-1.77	-3.08	-0.45	-2.33	-3.87	-0.80	-2.49	-3.96	-1.02	-3.09	-4.80	-1.39	0.13	-3.80	4.05

*Abbreviations: CI – confidence interval, EVAR – endovascular aortic repair, MRS – Marginal Rate of Substitution (*denominator AAA size (mm)).*

Attribute reference levels: Man, Anaesthetic risk – low, Anatomy on instructions for use (IFU), Patient wants procedure.

Supplemental References

1 de Bekker-Grob EW, Donkers B, Jonker MF, *et al.* Sample Size Requirements for Discrete-Choice Experiments in Healthcare: a Practical Guide. *The Patient - Patient-Centered Outcomes Research*. 2015;8:373–84. doi: 10.1007/s40271-015-0118-z

2 Lancaster KJ. A New Approach to Consumer Theory. *Journal of Political Economy*. 1966;74:132–57. doi: 10.1086/259131

3 Ryan M, Gerard K, Amaya-Amaya M, editors. *Using Discrete Choice Experiments to Value Health and Health Care*. Dordrecht: Springer Netherlands 2008.

4 Bridges JFP, Hauber AB, Marshall D, *et al.* Conjoint analysis applications in health--a checklist: a report of the ISPOR Good Research Practices for Conjoint Analysis Task Force. *Value Health*. 2011;14:403–13. doi: 10.1016/j.jval.2010.11.013

5 Lancsar E, Louviere J. Conducting Discrete Choice Experiments to Inform Healthcare Decision Making. *Pharmacoeconomics*. 2008;26:661–77. doi: 10.2165/00019053-200826080-00004

6 Wanhainen A, Verzini F, Herzeele I Van, *et al.* European Society for Vascular Surgery (ESVS) 2019 Clinical Practice Guidelines on the Management of Abdominal Aorto-iliac Artery Aneurysms. *European Journal of Vascular & Endovascular Surgery*. Published Online First: 2019. doi: 10.1016/j.ejvs.2018.09.020

7 Potgeiter R, Hindley H, Mitchell D, *et al.* Delivering a National Quality Improvement Programme for Patients with Abdominal Aortic Aneurysms. 2012.

8 *Abdominal aortic aneurysm : diagnosis and management Evidence review T: Effectiveness of endovascular aneurysm repair compared with open surgical repair of ruptured abdominal aortic aneurysms NICE*. 2020.

9 Khashram M. Systematic Review and Meta-analysis of Factors In fl uencing Survival Following Abdominal Aortic Aneurysm Repair. *European Journal of Vascular & Endovascular Surgery*. 2016;51:203–15. doi: 10.1016/j.ejvs.2015.09.007

10 Sutton R, Bann S, Brooks M, *et al.* The surgical risk scale as an improved tool for risk-adjusted analysis in comparative surgical audit. *British Journal of Surgery*. 2002;89:763–8. doi: 10.1046/j.1365-2168.2002.02080.x

11 Bertges DJ, Goodney PP, Zhao Y, *et al.* The Vascular Study Group of New England Cardiac Risk Index (VSG-CRI) predicts cardiac complications more accurately than the Revised Cardiac Risk Index in vascular surgery patients. *J Vasc Surg*. 2010;52:674-683.e3. doi: 10.1016/j.jvs.2010.03.031

12 Braun K, Brunkwall J, Gawenda M. Risikoeinschätzung in der Aortenchirurgie - Evaluation des SVS / AAVS Comorbidity Severity Score. *Zentralbl Chir*. 2007;132:477–84. doi: 10.1055/s-2007-981358

13 Karthikesalingam A, Vidal-Diez A, De Bruin JL, *et al.* International validation of a risk score for complications and reinterventions after endovascular aneurysm repair. *British Journal of Surgery*. 2015;102:509–15. doi: 10.1002/bjs.9758

14 Patterson BO, Holt PJE, Hinchliffe R, *et al.* Predicting Risk in Elective Abdominal Aortic Aneurysm Repair: A Systematic Review of Current Evidence. *European Journal of Vascular and Endovascular Surgery*. 2008;36:637–45. doi: 10.1016/j.ejvs.2008.08.016

15 Barnes M, Boult M, Maddern G, *et al.* A Model to Predict Outcomes for Endovascular Aneurysm Repair Using Preoperative Variables. *European Journal of Vascular and Endovascular Surgery*. 2008;35:571–9. doi: 10.1016/j.ejvs.2007.12.003

16 Kertai MD, Boersma E, Klein J, *et al.* Optimizing the Prediction of Perioperative Mortality in Vascular Surgery by Using a Customized Probability Model. *Arch Intern Med*. 2005;165:898. doi: 10.1001/archinte.165.8.898

17 Vanzetto G, Machecourt J, Boendea D, *et al.* Additive value of thallium single-photon emission computed tomography myocardial imaging for prediction of perioperative events in clinically selected high cardiac risk patients having abdominal aortic surgery. *Am J Cardiol*. 1996;77:143–8. doi: 10.1016/S0002-9149(96)90585-8

18 Eagle KA, Coley C, Newell J, *et al.* Combining Clinical and Thallium Data Optimizes Preoperative Assessment of Cardiac Risk before Major Vascular Surgery. *Ann Intern Med*. 1989;110:859. doi: 10.7326/0003-4819-110-11-859

19 Tang T, Walsh SR, Fanshawe TR, *et al.* Estimation of physiologic ability and surgical stress (E-PASS) as a predictor of immediate outcome after elective abdominal aortic aneurysm surgery. *The American Journal of Surgery*. 2007;194:176–82. doi: 10.1016/j.amjsurg.2006.10.032

20 Chaikof EL, Fillinger MF, Matsumura JS, *et al.* Identifying and grading factors that modify the outcome of endovascular aortic aneurysm repair. *J Vasc Surg*. 2002;35:1061–6. doi: 10.1067/mva.2002.123991

21 Steyerberg EW, Kievit J, de Mol Van Otterloo JC, *et al.* Perioperative mortality of elective abdominal aortic aneurysm surgery. A clinical prediction rule based on literature and individual patient data. *Arch Intern Med*. 1995;155:1998–2004.

22 Carlisle JB, Danjoux G, Kerr K, *et al.* Validation of long-term survival prediction for scheduled abdominal aortic aneurysm repair with an independent calculator using only pre-operative variables. *Anaesthesia*. 2015;70:654–65. doi: 10.1111/anae.13061

23 Biancari F, Hobo R, Juvonen T. Glasgow Aneurysm Score predicts survival after endovascular stenting of abdominal aortic aneurysm in patients from the EUROSTAR registry. *British Journal of Surgery*. 2006;93:191–4. doi: 10.1002/bjs.5262

24 Ambler GK, Gohel MS, Mitchell DC, *et al.* The Abdominal Aortic Aneurysm Statistically Corrected Operative Risk Evaluation (AAA SCORE) for predicting mortality after open and endovascular interventions. *J Vasc Surg*. 2015;61:35-43.e1. doi: 10.1016/j.jvs.2014.06.002

25 Giles KA, Schermerhorn ML, O'Malley AJ, *et al.* Risk prediction for perioperative mortality of endovascular vs open repair of abdominal aortic aneurysms using the Medicare population. *J Vasc Surg*. 2009;50:256–62. doi: 10.1016/j.jvs.2009.01.044

26 Grant SW, Grayson AD, Purkayastha D, *et al.* Logistic risk model for mortality following elective abdominal aortic aneurysm repair. *British Journal of Surgery*. 2011;98:652–8. doi: 10.1002/bjs.7463

27 Prytherch DR, Sutton GL, Boyle JR. Portsmouth POSSUM models for abdominal aortic aneurysm surgery. *British Journal of Surgery*. 2002;88:958–63. doi: 10.1046/j.0007-1323.2001.01820.x

28 Tang T, Walsh SR, Prytherch DR, *et al.* VBHOM, a data economic model for predicting the outcome after open abdominal aortic aneurysm surgery. *British Journal of Surgery*. 2007;94:717–21. doi: 10.1002/bjs.5808

29 Grant SW, Hickey GL, Grayson AD, *et al.* National risk prediction model for elective abdominal aortic aneurysm repair. *British Journal of Surgery*. 2013;100:645–53. doi: 10.1002/bjs.9047

30 Coast J, Horrocks S. Services Research & Policy Developing attributes and levels for discrete choice experiments using qualitative methods. *J Health Serv Res Policy*. 2007;12:25–30. doi: 10.1258/135581907779497602

31 Maxwell J. Understanding and Validity in Qualitative Research. *Harv Educ Rev*. 1992;62:279–301. doi: 10.17763/haer.62.3.8323320856251826

32 Qualitative methods for health research. *Choice Reviews Online*. 2009;47:47-0901-47–0901. doi: 10.5860/CHOICE.47-0901

33 Braun V, Clarke V. Using thematic analysis in psychology. *Qual Res Psychol*. 2006;3:77–101. doi: 10.1191/1478088706qp063oa

34 Braun V, Clarke V. Thematic analysis. *APA handbook of research methods in psychology, Vol 2: Research designs: Quantitative, qualitative, neuropsychological, and biological.* Washington: American Psychological Association 2012:57–71.

35 Varpio L, Paradis E, Uijtdehaage S, *et al.* The Distinctions Between Theory, Theoretical Framework, and Conceptual Framework. *Academic Medicine*. 2020;95:989–94. doi: 10.1097/ACM.0000000000003075

36 Deterding NM, Waters MC. Flexible Coding of In-depth Interviews: A Twenty-first-century Approach. *Sociol Methods Res*. 2021;50:708–39. doi: 10.1177/0049124118799377

37 Xue Q-L. The Frailty Syndrome: Definition and Natural History. *Clin Geriatr Med*. 2011;27:1–15. doi: 10.1016/j.cger.2010.08.009

38 Abiiro GA, Leppert G, Mbera GB, *et al.* Developing attributes and attribute-levels for a discrete choice experiment on micro health insurance in rural Malawi. *BMC Health Serv Res*. 2014;14:235. doi: 10.1186/1472-6963-14-235

39 Bekker-Grob EW, Donkers B, Bliemer MCJ, *et al.* Can healthcare choice be predicted using stated preference data ? *Soc Sci Med*. 2020;246:112736.

40 Hahn GJ, Shapiro SS, General Electric Company. *A catalog and computer program for the design and analysis of orthogonal symmetric and asymmetric fractional factorial experiments*. 1st ed. General Electric, Research and Development Center, Schenectady, New York, 1966 1966.

41 Day B, Bateman IJ, Carson RT, *et al.* Ordering effects and choice set awareness in repeat-response stated preference studies. *J Environ Econ Manage*. 2012;63:73–91. doi: 10.1016/j.jeem.2011.09.001

42 Scali ST, Arnaoutakis DJ, Huber TS, *et al.* The Influence of Surgeon Experience on Patient Selection and Outcomes After Open Abdominal Aortic Aneurysm Repair. *J Vasc Surg*. 2019;70:e48. doi: 10.1016/j.jvs.2019.06.074

43 Wallis CJD, Jerath A, Coburn N, *et al.* Association of Surgeon-Patient Sex Concordance With Postoperative Outcomes. *JAMA Surg*. 2022;157:146. doi: 10.1001/jamasurg.2021.6339

44 Boyle JR, Mao J, Beck AW, *et al.* Editor's Choice – Variation in Intact Abdominal Aortic Aneurysm Repair Outcomes by Country: Analysis of International Consortium of Vascular Registries 2010 – 2016. *European Journal of Vascular and Endovascular Surgery*. 2021;62:16–24. doi: 10.1016/j.ejvs.2021.03.034

45 Beck AW, Sedrakyan A, Mao J, *et al.* Variations in Abdominal Aortic Aneurysm Care: A Report From the International Consortium of Vascular Registries. *Circulation*. 2016;134:1948–58. doi: 10.1161/CIRCULATIONAHA.116.024870

46 Mariel P, Hoyos D, Meyerhoff J, *et al.* Experimental Design. 2021:37–49.

47 Orme B. Sample size issues for conjoint analysis studies. 1998.

48 RStudio Team (2020). RStudio: Integrated Development Environment for R. RStudio, PBC, Boston, MA. 2020.

49 Hess S, Palma D. Apollo: A flexible, powerful and customisable freeware package for choice model estimation and application. *Journal of Choice Modelling*. 2019;32:100170. doi: 10.1016/j.jocm.2019.100170

50 Daly A, Hess S, de Jong G. Calculating errors for measures derived from choice modelling estimates. *Transportation Research Part B: Methodological*. 2012;46:333–41. doi: 10.1016/j.trb.2011.10.008

51 Hess S, Train KE, Polak JW. On the use of a Modified Latin Hypercube Sampling (MLHS) method in the estimation of a Mixed Logit Model for vehicle

choice. *Transportation Research Part B: Methodological*. 2006;40:147–63. doi: 10.1016/j.trb.2004.10.005
