# Supplementary material for: Impact of patient sex on selection for abdominal aortic aneurysm repair: a discrete choice experiment
Source: BMJ Open. 2025 Feb 26;15(2):e091661. doi: 10.1136/bmjopen-2024-091661 (PMC11865737; doi:10.1136/bmjopen-2024-091661)
Supplement: Supplementary file 3 [file bmjopen-2024-091661.reviewer_comments.pdf]

PEER REVIEW HISTORY

BMJ Open publishes all reviews undertaken for accepted manuscripts. Reviewers are asked to complete a checklist review form and are provided with free text boxes to elaborate on their assessment. These free text comments are reproduced below.

ARTICLE DETAILS

Title (Provisional)

The Impact of Patient Sex on Selection for Abdominal Aortic Aneurysm Repair: A Discrete Choice Experiment

Authors

Pouncey, Anna Louise; Loría-Rebolledo, Luis Enrique; Sharples, Linda; Bicknell, Colin; Ryan, Mandy; Powell, Janet

---

VERSION 1 - REVIEW

**Reviewer**	**1**
**Name**	**Lindeman, Jan**
**Affiliation**	**Leiden University Medical Center, Vascular surgery**
**Date**	**12-Aug-2024**
**COI**	**None**

The study by Pouncey and colleagues is interesting as it provides quantitative insight in the surgeon's decision making process. Startled by the alarming introductionary statement that "Despite a four-fold higher risk of rupture at smaller abdominal aortic aneurysm (AAA) diameters for women compared with men, previous research indicates that women with an AAA are 25% less likely to be offered elective surgical repair", and its weak underpinning (reference 2 is based on numerous assumptions and questionable statistics) I performed a small literature review and found the following proportions for the # elective over emergency procedures for women: Aus 1.18; DK 0.97; Fin 1.40; Hun 1.06; NZ 0.93; NOR 1.25; SWE 1.43; SWZ 1.29; UK 1.56; NL 1.07 (data extracted from Eur J Vasc Endovasc Surg. 2024; 68: 162-170 and J Vasc Surg. 2018; 67: 735-739. These data do not support the dramatic claims in the introduction, and the data suggest that the claimed sex-disparities differ per country and may, at least in part relate to the existence or absence of AAA screening programs for men. In this respect I think that the paper requires a fundamental overhaul with more realistic backgroud information on the rationale.

No comments with respect to methods and results

Discussion: see comments on the introduction.

Pg21, L36 "On interaction analyses age was not observed to have a significant effect for men, but had a detrimental association for women" where is this conclusion based on, it could well reflect a balanced decision based on an assumed high procedural risk.

P21, L44: women less comorbidity: other studies have shown the reverse: Ann Surg. 2023; 278: 815-822.

**Reviewer**	**2**
**Name**	**Eid, Mark**
**Affiliation**	**Dartmouth-Hitchcock Medical Center, General Surgery**
**Date**	**06-Nov-2024**
**COI**	**none**

The authors sought to study which factors influence surgeon preference of choice of AAA repair, particularly among women patients. I commend the authors' efforts, utilizing a unique study design to shed light on this important topic.

Overall, the manuscript is well designed. Please be mindful of grammatical errors throughout the manuscript and edit as such .

General comments:

the typical readers are not going to be familiar with your study design and methodology - this is your opportunity to teach them- As such, I would recommend that the methods section needs to be expanded on, with less reliance on supplemental material as the location of such information.

- For instance, the first paragraph of your methods describes multiple theories which lay the foundation for DCE- if this information is truly important, it must be expanded upon.

Table 1: the names of variables that were used in the analysis (i.e. sex0/sex1) do not need to be reported in the table- this table would be more appropriate as a supplement, as its not a true result.

Analysis: In your description of your econometric analysis- please explicitly state what is the dependent and independent variables of interest and the research question as to guide the reader.

the entirety of page 13 belongs in supplement

Results

page 15 lines 30-38- would recommend revision as there is a long run-on sentence, and the information presented is not clear. There is also reference to conclusions in the results, which I would recommend to refrain.

In terms of how your results were displayed, the tables are not easily followed and It would be better to to be displayed in a forest plot. The statistical analysis is unique and quite complex- I recommend that a statistical analyst review the results.

Discussion

Multiple paragraphs in the discussion discuss the limitations of the manuscript. While I commend the authors' candidness, please consolidate to a single "limitations paragraph. I would also recommend to organize the discussion as detailed by the manuscript entitled "Preparing Manuscripts for Submission to Medical Journals: The Paper Trail" by Welch.

Please edit language to be more direct- i.e. page 21 line 58, some of the results are referenced as " a greater than average percentage..." This language is not clear and hard to follow. Also the sentence "Preference did however vary..." on page 21, line 54 could more simply be stated as " more experienced surgeons were more likely to offer surgical repair to older patients." Not only is this more clear , it also allows the author the ability to then elaborate on the result, referencing previous data and postulating a conclusion to this point. I would recommend that the authors do this for all of their results in the discussion.

Please also add a conclusion paragraph

**Reviewer**	**3**
**Name**	**Dack, Kyle**
**Affiliation**	**University of Bristol, MRC Integrative Epidemiology Unit**
**Date**	**20-Nov-2024**
**COI**	**None**

This paper reports a novel method of analysis and results from a well designed experiment. I recommend revisions primarily because it is missing (a) a lay explanation of what the units of analysis (MRS) means and (b) essential details regarding the modelling strategy. I am also concerned that adjustment to minimize confounding bias is mentioned in the abstract but I see no information about it in the main paper.

Abstract

1. The “intervention” section repeats the same information as “design”. It would be more useful if it explained how the experimental conditions varied.

2. I understand marginal rate of substitution in terms of willingness to pay. But it is not immediately clear to me (and possibly other readers) what an MRS of “3.86” means in the context of a surgeon’s decision. This is not a common unit of analysis in health science, so it would be very helpful to have a sentence explaining in lay terms what 3.86 means in practical terms.

3. P4 line 23: “Adjusting for major confounders, a woman patient was more likely to be offered AAA repair (marginal rate of substitution (MRS) 3.86 [2.93, 4.79])”

4. P5 line 21: “Adjusting for major confounders, women with an AAA were not less likely to be offered surgical repair.” –While true, it would be more precise to state that they were more likely to be offered repair, as reported in the abstract.

Introduction

5. -The aims are clear and well justified, no suggestions.

Methods

6. Page 10/11: The sentence between these two pages overlaps.

7. Table 1. I don’t think the “name” column adds anything to this as it is just the variable codes, which do not seem to be used anywhere in the manuscript.

8. Table 1 is not referenced in the text. Before presenting it, please explain what is meant by indirect utility. A formula is provided but this is shown after table 1, and does not include a lay explanation. At the start of the methods there is a good summary of the DCE method – I suggest adding to this a summary of what the effect estimate is intended to represent.

9. Please add the reason why continuous data was centered / scaled. A requirement of the analysis method or to aid with a certain interpretation?

10. “Surgeons’ choice data were first analysed using logistic regression models” What were the models? It would help to begin this section by explaining the modelling strategy, i.e. how many models, and whether exposures were all modelled together or if a different adjustment set was used for each exposure/outcome relationship.

11. “Pre-specified subgroup analyses were conducted to assess preference heterogeneity associated with surgeon characteristics” please list these.

12. “Models were compared using likelihood ratio chi-square statistic and Akaike Information Criterion and goodness of fit was estimated using adjusted pseudo R2” Which models were compared? Please explain how these comparisons were used.

13. The abstract reported likelihood of repair decisions adjusted for confounders. But in the methods there are no details of what confounding factors were identified, and how these were adjusted for. E.g. the same adjustment set used for modelling AAA repair on patient sex and AAA repair on anesthetic risk. Information is missing on what models included which variables.

Results

14. P15 line 36. Please add something like this to the abstract and early in the methods because it is critical to understanding the analysis.

15. All tables: (a) Please replace the “estimate” column names with the units, which I think are “odds ratio of repair” and “MRS”. (b) Its not clear whether the model fit statistics were used for anything – if not then I suggest removing. (c) Please make the number of decimal places consistent in all estimates (d) Some p-values are reported as zero.

16. Table 2: (a) This is currently labelled as table 1.

17. It looks as if all factors of interest were modelled together against the outcome decision. This involves an assumption that all are suitable covariates to adjust for confounding, i.e. “age and surgical decision” is confounded by the same factors as “Patient anxiety and surgical decision”. There is also the risk that factors could be on the same causal pathway, and the effect of interest is being split. There should at least be an explanation of the approach to confounding in the methods and any limitations (unmeasured confounding factors) in the discussion.

18. The section concerning respondent heterogeneity reports predicted vs observed decisions. Please explain how this was generated in the methods section – I cannot find details of it?

Discussion

19. The abstract and discussion state “Patient sex alone, did not account for real-world disparity observed in selection for surgery.” and “patient sex alone, does not account for the observed disparity in overall clinician selection process”, respectively. I think this means that the experimental results are not consistent with real world observations, because the former find women were more likely to be offered surgery, while the latter finds women are less likely. If this is the intended meaning I suggest stating this more directly, because it is hard to understand the meaning of “does not account for” in this context.

20. Please add a section on strengths and limitations, including an assessment of the risk of confounding (residual or latent).

VERSION 1 - AUTHOR RESPONSE

Reviewer: 1
Dr. Jan Lindeman, Leiden University Medical Center

Comments to the Author:
The study by Pouncey and colleagues is interesting as it provides quantitative insight in the surgeon's decision making process. Startled by the alarming introductionary statement that "Despite a four-fold higher risk of rupture at smaller abdominal aortic

aneurysm (AAA) diameters for women compared with men, previous research indicates that women with an AAA are 25% less likely to be offered elective surgical repair", and its weak underpinning (reference 2 is based on numerous assumptions and questionable statistics)

Both references are from reputable peer reviewed journals (The Lancet and JVS) and appropriately cited. We have added the following further references with regards to increased rupture rates for women.

Surveillance Intervals for Small Abdominal Aortic Aneurysms: A Meta-analysis The RESCAN Collaborators*JAMA (2013) 309(8) 806-813*

Sweeting MJ, Thompson SG, Brown LC, *et al*. Meta-analysis of individual patient data to examine factors affecting growth and rupture of small abdominal aortic aneurysms. *British Journal of Surgery*. 2012;99:655–65. doi: 10.1002/bjs.8707

I performed a small literature review and found the following proportions for the # elective over emergency procedures for women: Aus 1.18; DK 0.97; Fin 1.40; Hun 1.06; NZ 0.93; NOR 1.25; SWE 1.43; SWZ 1.29; UK 1.56; NL 1.07 (data extracted from Eur J Vasc Endovasc Surg. 2024; 68: 162-170 and J Vasc Surg. 2018; 67: 735-739. These data do not support the dramatic claims in the introduction, and the data suggest that the claimed sex-disparities differ per country and may, at least in part relate to the existence or absence of AAA screening programs for men.

The effect of screening is already considered and referenced as follows in the introduction: "In the UK, this disparity in surgical selection is compounded by the availability of population-based AAA screening for men alone[6]. As a result, although one fifth of AAAs diagnoses and one third of AAA ruptures are in women, only one tenth of elective repairs are undertaken in women[1,7,8]."

Registries do not show how many women are turned down for emergency repair, this only comes via national data linkage and was not performed in all countries cited by the reviewer. We would like to clarify that women are also less likely to receive an emergency repair than men, as clearly demonstrated within the following citation, which renders examination of proportions of elective:emergency cases inaccurate for assessment of morbidity/mortality burden:
Maheswaran R, Tong T, Michaels J, et al. Socioeconomic disparities in abdominal aortic aneurysm repair rates and survival. British Journal of Surgery. 2022;109:958–67. doi: 10.1093/bjs/znac222

In this respect I think that the paper requires a fundamental overhaul with more realistic background information on the rationale.

We politely disagree. Data within the introduction are appropriately cited and provide a clear rationale for the research conducted. The study was conducted with equipoise and adds to knowledge on the topic.

We have however, extensively re-written the paper in response to all reviewers comments and believe that it is now contains appropriate lay explanations and is more accessible for the target audience.

No comments with respect to methods and results

Discussion: see comments on the introduction.
Pg21, L36 "On interaction analyses age was not observed to have a significant effect for men, but had a detrimental association for women" where is this conclusion based on,

Please see Results section and Table 4. Error component model for AAA repair versus no repair, with patient sex-age interaction term.

it could well reflect a balanced decision based on an assumed high procedural risk.
P21, L44: women less comorbidity: other studies have shown the reverse: Ann Surg. 2023; 278: 815-822.

Specifically, the DCE design is unique in that it allows use to quantify the indirect utility of attributes such as age and sex, while adjusting for differences in other presenting factors such as co-morbid status etc.

Whether decisions made by surgeons are justified or not is beyond the scope of this manuscript. We only present an analysis of the relative importance of patient attributes which influence surgeon's decision making.

We do however have the following within the discussion which we believe covers the points addressed:

"Given the lack of prognostic data for women with AAA, surgeons may rely on sex stereotypes, or personal experience to make operative decisions. A similar tendancy toward less invasive treatment has been reported in cardiology, where women are less likely to receive complete revascularisation, and considerably less likely to receive a coronary artery bypass grafting[99,100]. Due to the lack of AAA screening for women, their AAAs are more often detected incidentally and their risk factors are less likely to be controlled. Combined with increased elective mortality rates, this may lead vascular surgeons to favour less-invasive options[88,101]. Additionally, the sex-frailty paradox - where women more often meet frailty criteria, but experience lower mortality rates than men - may further increase preference for less invasive repair[102]. However, these tendencies may also arise from a form of "benevolent sexism", or "protective paternalism", where a desire to protect women, inadvertently results in a lower standard of care[103,104]."

Reviewer: 2
Dr. Mark Eid, Dartmouth-Hitchcock Medical Center, White River Junction VA Medical Center

Comments to the Author:
The authors sought to study which factors influence surgeon preference of choice of AAA repair, particularly among women patients. I commend the authors' efforts, utilizing a unique study design to shed light on this important topic.
Overall, the manuscript is well designed. Please be mindful of grammatical errors throughout the manuscript and edit as such .

Many thanks for your review and kind comments.
We have proofread and adjusted accordingly.

General comments:
the typical readers are not going to be familiar with your study design and methodology - this is your opportunity to teach them- As such, I would recommend that the methods section needs to be expanded on, with less reliance on supplemental material as the location of such information.
- For instance, the first paragraph of your methods describes multiple theories which lay the foundation for DCE- if this information is truly important, it must be expanded upon.

Many thanks. We have adjusted the text accordingly.

Table 1: the names of variables that were used in the analysis (i.e. sex0/sex1) do not need to be reported in the table- this table would be more appropriate as a supplement, as its not a true result.

We have elected to keep the table in the manuscript as we feel it provides key oversight regarding the structure of the discrete choice experiment, aiding the reader's understanding, but have deleted the redundant "name column" as suggested.

Analysis: In your description of your econometric analysis- please explicitly state what is the dependent and independent variables of interest and the research question as to guide the reader.

We have extensively re-worked the text to explain the research questions more explicitly

"To evaluate vascular surgeons' decisions regarding (A) AAA repair and (B) selection for EVAR a two-tier DCE was designed. The experiment presented a series of case scenarios featuring AAA patients with varying attributes. Surgeons were asked to make two forced choices for each scenario: (A) AAA repair decision: would you offer this patient AAA repair? (Yes/No); (B) repair method decision: assuming this patient has been deemed suitable for repair, and could have an infrarenal clamp, would you prefer to offer endovascular rather than open repair? (Yes/No)."

We have also added a more readable and comprehensive explanation of the utility function and econometric analysis. See comment 8 of reviewer 3.

the entirety of page 13 belongs in supplement

We have condensed the utility function within the methods to increase readability, but believe that it is important for understanding of the methodology, by most readers, to retain some description of it within the text . See comment 8 of reviewer 3.

Results
page 15 lines 30-38- would recommend revision as there is a long run-on sentence, and the information presented is not clear. There is also reference to conclusions in the results, which I would recommend to refrain.

Many thanks, we have amended the entire text, including the results section, to increase readability.

In terms of how your results were displayed, the tables are not easily followed and It would be better to to be displayed in a forest plot. The statistical analysis is unique and quite complex- I recommend that a statistical analyst review the results.

Figures 2-5 demonstrate the relevant econometric results in visual format.
We have ensured that our authorship includes experts in DCE conduct and methodology (LELR and MR). They have personally reviewed the raw data, code and results.

Discussion
Multiple paragraphs in the discussion discuss the limitations of the manuscript. While I commend the authors' candidness, please consolidate to a single "limitations paragraph. I would also recommend to organize the discussion as detailed by the manuscript entitled "Preparing Manuscripts for Submission to Medical Journals: The Paper Trail" by Welch.

Many thanks, we have consolidated these paragraphs and restructured the discussion to meet BMJ Open requirements.

Please edit language to be more direct- i.e. page 21 line 58, some of the results are referenced as " a greater than average percentage..." This language is not clear and hard to follow. Also the sentence "Preference did however vary..." on page 21, line 54 could more simply be stated as " more experienced surgeons were more likely to offer surgical repair to older patients." Not only is this more clear , it also allows the author the ability to then elaborate on the result, referencing previous data and postulating a conclusion to this point. I would recommend that the authors do this for all of their results in the discussion.

Many thanks, we have adjusted the text to increase clarity as suggested.

Please also add a conclusion paragraph

Many thanks, we have added as requested.

Reviewer: 3
Dr. Kyle Dack, University of Bristol

Comments to the Author:
This paper reports a novel method of analysis and results from a well designed experiment. I recommend revisions primarily because it is missing (a) a lay explanation of what the units of analysis (MRS) means and (b) essential details regarding the modelling strategy. I am also concerned that adjustment to minimize confounding bias is mentioned in the abstract but I see no information about it in the main paper.

Many thanks for your insightful comment. We have addressed each of these points, as outlined below and believe we have substantially improved the readability of the paper.

Abstract
1. The “intervention” section repeats the same information as “design”. It would be more useful if it explained how the experimental conditions varied.

We have adjusted as follows:

Setting: Simulated environment using case scenarios with varying patient attributes.
Participants: Vascular Surgeons
Interventions: Selection for infrarenal AAA repair
Main Outcome Measures: (A) AAA repair versus no repair, and (B) endovascular versus open repair.

2. I understand marginal rate of substitution in terms of willingness to pay. But it is not immediately clear to me (and possibly other readers) what an MRS of “3.86” means in the context of a surgeon’s decision. This is not a common unit of analysis in health science, so it would be very helpful to have a sentence explaining in lay terms what 3.86 means in practical terms.

We have added the following:

“To further aid interpretation, marginal rates of substitution (MRS), which enable assessment of trade-off between attributes, were calculated using AAA size (mm) as the denominator and the delta method for standard error estimation[83]. For instance, if a surgeon is deciding whether to offer a procedure based on a patient's age and the size of their aneurysm, the MRS would indicate how much of an increase in AAA size would be needed to offset an increase in the patient's age in order to keep the likelihood of offering the procedure the same.”

3. P4 line 23: “Adjusting for major confounders, a woman patient was more likely to be offered AAA repair (marginal rate of substitution (MRS) 3.86 [2.93, 4.79])”
4. P5 line 21: “Adjusting for major confounders, women with an AAA were not less likely to be offered surgical repair.” –While true, it would be more precise to state that they were more likely to be offered repair, as reported in the abstract.

Many thanks, this section has subsequently been removed from the text as per Editor's request above.

Introduction
5. -The aims are clear and well justified, no suggestions.

Many thanks

Methods
6. Page 10/11: The sentence between these two pages overlaps.

We think this was a formatting error. The manuscript has been revised and proofread accordingly.

7. Table 1. I don't think the "name" column adds anything to this as it is just the variable codes, which do not seem to be used anywhere in the manuscript.

Many thanks, we have removed this redundant column.

8. Table 1 is not referenced in the text. Before presenting it, please explain what is meant by indirect utility.

We have removed reference to indirect utility in the main paper as this technical terminology is not necessary for understanding of the paper. We have also ensured that Table 1 is referenced in the text: "The final selected attributes and levels are presented in **Error! Reference source not found.**."

A formula is provided but this is shown after table 1, and does not include a lay explanation. At the start of the methods there is a good summary of the DCE method – I suggest adding to this a summary of what the effect estimate is intended to represent.

We have changed the text to provide clear explanation of the utility function with explanation of the effect estimate as follows:

"Separate models were constructed for each outcome: (A) the likelihood of selecting AAA repair versus turndown and (B) the likelihood of selecting EVAR versus open repair. Each model included all relevant exposure variables: patient sex, age, aneurysm size, anatomical suitability, anaesthetic risk and patient perspective. The following utility/benefit function was estimated for each analysis using an error components model.

$V_{surgical\_repair/endovascular\_repair} = ASC_0 + \beta_1 female\_sex + \beta_2 age + \beta_3 anaes\_mod + \beta_4 anaes\_high + \beta_5 anaes\_vhigh + \beta_6 patient\_anxiety + \beta_7 AAA\_diameter + \beta_8 anatomy\_neck + \beta_9 anatomy\_access + \beta_{10} anatomy\_neck{+}access$

$V_{turndown/open\_repair} = ASC_1$

Within this utility function, V represents the systematic utility —the predictable, measurable component of a decision based on known factors. The reference levels are “turndown” for analysis (A) and “open repair” for analysis (B). The error components model was chosen to relax of the irrelevance of independent alternatives assumption, classifying alternative specific constants as random: ASC_0 is set to 0 while ASC_1, which captures the unexplained variation in choice for the reference levels, is modelled as a normally distributed random parameter, and estimated using 500 Modified Latin Hypercube Sampling inter-individual draws[82].

Within the function, $\beta_{1\text{-}10}$ represent specific parameter coefficients associated with the defined attribute levels. Because utility is on a relative scale, the coefficients are interpreted in relation to each other rather than in absolute terms. A positive value indicates that the attribute level combination was preferred relative to the reference level and a larger value indicates a stronger preference. To further aid interpretation, marginal rates of substitution (MRS), which enable assessment of trade-off between attributes, were calculated using AAA size (mm) as the denominator and the delta method for standard error estimation[83]. For instance, if a surgeon is deciding whether to offer a procedure based on a patient's age and the size of their aneurysm, the MRS would indicate how much of an increase in AAA size would be needed to offset an increase in the patient's age in order to keep the likelihood of offering the procedure the same. The mean percentage difference in the probability of selection for women and men were also calculated for a variety of scenarios.”

9. Please add the reason why continuous data was centered / scaled. A requirement of the analysis method or to aid with a certain interpretation?

The decision to centre and scale the continuous data was to help improve model interpretability, reduce multicollinearity, ensure model stability, and enhance the accuracy of error component estimation.

10. “Surgeons’ choice data were first analysed using logistic regression models” What were the models? It would help to begin this section by explaining the modelling strategy, i.e. how many models, and whether exposures were all modelled together or if a different adjustment set was used for each exposure/outcome relationship.

Within normal econometric analyses, it is standard to build your model from the most simple option first. We have added further explanation regarding the models used as above and have added the following regarding rationale for selection of an error component model.

“The error component model was chosen to allow relaxation of the IIA (irrelevance of independent alternatives assumption), classifying alternative specific constants as random:”

11. “Pre-specified subgroup analyses were conducted to assess preference

heterogeneity associated with surgeon characteristics" please list these.

Many thanks – we have moved the following text from the supplement to the main text for clarification:

Pre-specified subgroup analyses were conducted to assess preference heterogeneity associated with surgeon characteristics as follows: surgeon gender (men or women), level of experience (early: <10 years, mid-level: 10-20 years or experienced: >20 years), preference for EVAR repair (categorised into 1st "low", 2/3rd "mid-range" and 4th "high" quartiles) and geographical location (categorised as UK, Europe, North America or other).

12. "Models were compared using likelihood ratio chi-square statistic and Akaike Information Criterion and goodness of fit was estimated using adjusted pseudo R2" Which models were compared? Please explain how these comparisons were used.

We have clarified that this was for the assessment of model performance on introduction of interaction terms.

For each error component model, interactions between patient sex and other attributes were also introduced. Performance of models with interaction terms were compared using likelihood ratio chi-square statistic and Akaike Information Criterion and goodness of fit was estimated using adjusted pseudo R^2.

13. The abstract reported likelihood of repair decisions adjusted for confounders. But in the methods there are no details of what confounding factors were identified, and how these were adjusted for. E.g. the same adjustment set used for modelling AAA repair on patient sex and AAA repair on anesthetic risk. Information is missing on what models included which variables.

We apologise that this confusion likely arose due to a lack of clarity within our methods section. Both error components models used the utility function specified. By confounding factors we meant to reference other attributes specified within the choice set scenarios, as essentially this a simulated environment with restricted variables. We have altered the abstract as follows:

"When all other attributes were equal, a woman patient was more likely to be offered AAA repair"

Results

14. P15 line 36. Please add something like this to the abstract and early in the methods because it is critical to understanding the analysis.

We were unable to ascertain what this comment is referring to but hope that the changes to the abstract and methods to aid in clarification are sufficient.

15. All tables: (a) Please replace the "estimate" column names with the units, which I think are "odds ratio of repair" and "MRS".

Utility is a relative measure of preference and does not have a physical unit.

We believe this confusion has arisen due to us needing to further clarify the methodology within the manuscript. We have added the following:

“Because utility is on a relative scale, the coefficients are interpreted in relation to each other rather than in absolute terms. A positive value indicates that the attribute level combination was preferred relative to the reference level and a larger value indicates a stronger preference.”

(b) Its not clear whether the model fit statistics were used for anything – if not then I suggest removing.

We have removed as requested.

(c) Please make the number of decimal places consistent in all estimates (d) Some p-values are reported as zero.

Many thanks, we have adjusted all estimates to 2 dp and p values to 3 dp.

16. Table 2: (a) This is currently labelled as table 1.

We are sorry, we could not find this error? We have double checked the manuscript to ensure that all tables are labelled correctly.

17. It looks as if all factors of interest were modelled together against the outcome decision. This involves an assumption that all are suitable covariates to adjust for confounding, i.e. “age and surgical decision” is confounded by the same factors as “Patient anxiety and surgical decision”. There is also the risk that factors could be on the same causal pathway, and the effect of interest is being split. There should at least be an explanation of the approach to confounding in the methods and any limitations (unmeasured confounding factors) in the discussion.

The utility model adjusts for confounders by including multiple attributes (e.g., sex, age, AAA diameter, anaesthesia risk) in the systematic utility function, allowing the effect of each attribute to be estimated while holding others constant. The coefficients reflect these adjusted effects, reducing the risk of confounding. Alternative Specific Constants (ASCs) capture unobserved variation between alternatives, and when treated as random parameters, they account for baseline differences and individual-level heterogeneity. This combination of attributes and ASCs helps control for both measured and unmeasured confounders.

18. The section concerning respondent heterogeneity reports predicted vs observed decisions. Please explain how this was generated in the methods section – I cannot find details of it?

“Pre-specified subgroup analyses were also conducted to assess preference heterogeneity associated with surgeon characteristics (e.g., surgeon gender, level of experience, preference for EVAR repair and geographical location) reporting the proportion of observed choices compared to model predictions.”

Discussion
19. The abstract and discussion state “Patient sex alone, did not account for real-world disparity observed in selection for surgery.” and “patient sex alone, does not account for the observed disparity in overall clinician selection process”, respectively. I think this means that the experimental results are not consistent with real world observations, because the former find women were more likely to be offered surgery, while the latter finds women are less likely. If this is the intended meaning I suggest stating this more directly, because it is hard to understand the meaning of “does not account for” in this context.

We have changed the wording to:

“When all other attributes were equal, a woman was more likely to be offered an AAA repair”.

Typically women present at about 4 years older, have slightly smaller aneurysms, more often have complex anatomical features and/or respiratory disease:

“When all other attributes were equal, women were significantly more likely to be offered AAA repair (percentage difference (%) 25.40 [95%CI 3.84,46.96]), however, adjusting for real world differences such as an increase in age of 4 years for women, (21.50 [-1.44,44.45]), or for hostile anatomy (4.24 [-35.45, 43.93]), the selection advantage for women was no longer significant. Moreover, adjusting for high anaesthetic risk (-3.25 [-42.93, 36.44]) or for an aneurysm 5mm smaller in diameter (-8.18 [-37.31, 20.96]), women were less likely to be offered repair. A woman who was 4 years older, with hostile anatomy, high anaesthetic risk and a smaller aneurysm size (-5mm) was 43.88% [-99.83,12.08] less likely to be offered repair, compared to a man, with low anaesthetic risk, and anatomy within IFU. (See Figure 4.)”

Therefore, we have demonstrated that it is not sex, but associated attributes which contribute to disparities in repair rates observed.

20. Please add a section on strengths and limitations, including an assessment of the risk of confounding (residual or latent).

We have condensed the paragraphs on strengths and limitations within the discussion to sign post more clearly where this is being discussed.

Reviewer: 1

Competing interests: None

Reviewer: 2
Competing interests: none

Reviewer: 3
Competing interests: None

VERSION 2 - REVIEW

**Reviewer**	**3**
**Name**	**Dack, Kyle**
**Affiliation**	**University of Bristol, MRC Integrative Epidemiology Unit**
**Date**	**25-Dec-2024**
**COI**	

The authors have made considerable revisions to improve the clarity and thoroughness of how the methods and results are reported. The revised paper is much more accessible to a lay audience, giving good explanations and avoiding unnecessary technical terminology. My original review contained 20 items of feedback and all have been addressed.

Having re-read the manuscript I would recommend it for publication. I note that there is no publishing checklist, but I am unsure which would be appropriate in this case, because it is neither observational nor an RCT?
